# AAV NRF2 Gene Therapy Preserves Retinal Structure and Function in Rodent Models of Oxidative Damage

**DOI:** 10.1016/j.ymthe.2026.02.005

**Published:** 2026-02-06

**Authors:** Apolonia Gardner, Shuai Wang, Adam Daniels, Dan Li, Christine Wu, Lucas Lin, Christin Hong, Sophia R. Zhao, Kamil Kruczek, Genevieve Weist, Laura Barrio Real, Joan Wicks, Mohamad Nayal, Ashley Carter, Christine Ott, Virginia Haurigot, Richard T. Born, Constance L. Cepko

**Affiliations:** 1. Department of Genetics, Harvard Medical School, Boston, MA 02115, USA.; 2. Department of Ophthalmology, Harvard Medical School, Boston, MA 02115, USA.; 3. Howard Hughes Medical Institute, Chevy Chase, MD 20815, USA.; 4. Program in Virology, Harvard Medical School, Boston, MA 02115, USA.; 5. Gene Therapy Research, Spark Therapeutics, Philadelphia, PA 19104, USA.; 6. Department of Neurobiology, Harvard Medical School, Boston, MA 02115, USA.

**Keywords:** Dry AMD, oxidative stress, sodium iodate model, gene therapy, retinal pigment epithelium, adeno-associated virus, subretinal injection, NRF2

## Abstract

Dry age-related macular degeneration is the most frequent cause of visual impairment in individuals over age 50 in developed countries. It is characterized by deposits of oxidized proteins and lipids and results in progressive loss of high acuity vision. One major risk factor is smoking, which causes oxidative stress in many tissues, including the eye. We previously showed that an adeno-associated viral vector expressing human NRF2 (AAV8/Best1-NRF2), a transcription factor that regulates responses to oxidative damage, slowed degeneration in mouse models of another blinding disorder, retinitis pigmentosa, which also includes oxidative stress. Here, our AAV8/Best1-NRF2 vector was tested in a model of oxidative stress wherein sodium iodate was injected systemically, as this is often used to model dry age-related macular degeneration. Sodium iodate causes acute oxidative damage to the retinal pigment epithelial cells, which provide support to the photoreceptor cells. In addition, this toxin ultimately leads to photoreceptor death. Subretinal injection of AAV8/Best1-NRF2 led to protection of the retinal pigment epithelium and photoreceptors, as well as preservation of visual function, in rat and mouse sodium iodate models. AAV8/Best1-NRF2 may serve as an effective gene-agnostic therapy for diseases with oxidative stress, including dry age-related macular degeneration.

## Abstract (summary)

Oxidative stress occurs in diseases that lead to vision loss, including age-related macular degeneration (AMD). AMD is commonly modeled by injection of sodium iodate (NaIO_3_), an oxidizing agent. AAV-mediated delivery of NRF2, a transcription factor that regulates oxidative stress, protects the eye from NaIO_3_ damage in rat and mouse models.

## Graphical Abstract

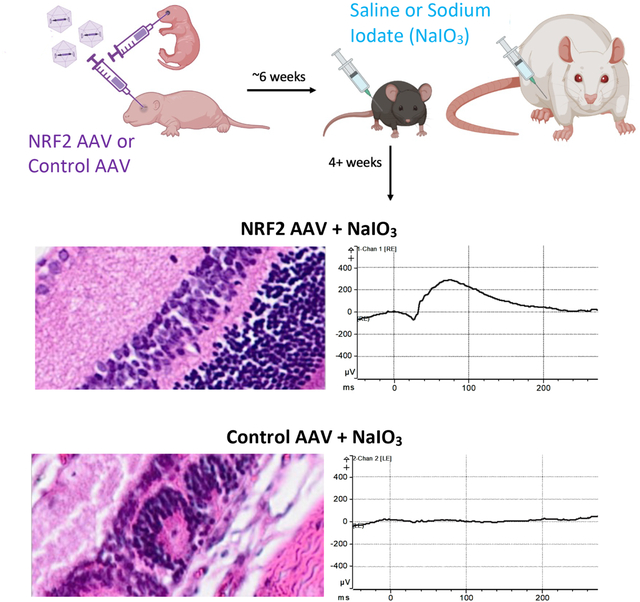

## Introduction

Age-related macular degeneration (AMD) is a disease leading to loss of high acuity vision, primarily in individuals over 50 years of age.^1^ There are two forms of AMD, dry and wet, where dry AMD comprises ~90% of all AMD cases.^2–5^ A hallmark symptom of AMD is the appearance of drusen, which are deposits between the retinal pigment epithelium (RPE) and an adjacent layer, Bruch’s membrane. These deposits are comprised of oxidized proteins and lipids.^6^ They form preferentially in the macula, an area of the retina surrounding the fovea, which is responsible for high acuity vision.^7,8^ Risk factors include genetic susceptibility, aging, lifestyle (obesity, diet, and smoking), and others.^9,10^ The initial symptoms, in some cases, are loss of choroidal vessels^11^ and/or loss of rod-mediated dark adaptation.^12^ Eventually, there is loss of RPE cells.^1,13^ RPE cells have several roles, including transport of nutrients from the choroidal vasculature to photoreceptors and the removal of waste by phagocytosing shed photoreceptor outer segments.^14^ RPE loss in patients with dry AMD generally transitions from small to progressively larger patches over many years. Geographic atrophy (GA) occurs when substantial areas of the RPE are lost in advanced stages of the disease.^15,16^ Wet AMD, a less common and more severe form of AMD, can occur in advanced stages of dry AMD or arise independently.^8^ Unlike dry AMD, “wet” AMD is so named because vasculature in the eye typically enlarges and hemorrhages, damaging the retina and obstructing vision.^3^ FDA-approved angiogenesis inhibitors (e.g., VEGF inhibitors^2^) are available and effective for wet AMD, and there are two FDA-approved complement inhibitors (pegcetacoplan and avacincaptad) to treat GA associated with dry AMD.^17^ Clinical trials with complement inhibitors showed that, while the rate of GA expansion was slowed, no improvement in visual acuity or daily functional vision was observed after one or two years of treatment, a factor that contributed to the European Medicines Agency (EMA) not approving the drug for dry AMD.^18^ Nutritional supplements, such as the AREDS^19^ and AREDS2 formulations,^20^ which contain antioxidants, represent an alternative treatment strategy for dry AMD by directly targeting oxidative stress, a believed driver of disease pathogenesis. In the AREDS clinical trial, patients showed a ~25% reduction in the risk of progression from intermediate to advanced AMD after taking this formulation.^19^ Albeit promising, the potency of this nutritional supplementation is low, leaving room for additional treatments to improve patient outcomes.

Many age-related diseases, including AMD, are thought to be caused, at least in part, by oxidative stress-induced damage.^21,22^ The eye is thought to be particularly susceptible to oxidative damage due to the high oxygen demands of photoreceptors and the high light environment.^23^ We previously showed that delivery of a transcription factor, NRF2, reduced the loss of cone photoreceptors, protected the RPE, and prolonged vision in mouse models of another retinal degenerative disease, retinitis pigmentosa.^24,25^ This therapy was effective in 3 mouse models of retinitis pigmentosa (3 different genotypes), potentially making it a disease gene-agnostic therapy. NRF2 delivery was achieved using adeno-associated virus (AAV), a gene therapy vector that is the vector of choice for ocular diseases.^26^ AAV vectors are favorable for *in vivo* applications due to their nonpathogenicity, broad tissue tropism, high titer, and stable episomal genome in non-dividing cells.^27^ In addition, AAV vectors can be engineered to express a gene specifically in the RPE or photoreceptors.

NRF2 is a transcription factor that undergoes proteasomal degradation in the cytoplasm under homeostatic conditions, but upon oxidative or xenobiotic stress, translocates to the nucleus and activates target genes to combat the effects of oxidative stress.^24,25,28,29^ A number of studies have tested the ability of NRF2 to reduce stress-related phenotypes or pathological symptoms in several diseases.^30^ Nrf2 levels can be raised by targeting its interaction with Keap1,^31^ the protein that directs its ubiquitination and proteasomal degradation, e.g. by mutating amino acids on Nrf2 that mediate the Keap1 interaction,^32^ or by genetic deletion of Keap1.^33^ There are also dietary compounds touted to be beneficial in aging and a variety of diseases, with effects thought to be mediated by NRF2.^34,35^ Treatment with a small molecule, such as dimethyl fumarate, as is done for multiple sclerosis, is also thought to increase NRF2 activity.^36^ In addition to fighting oxidative damage, NRF2 might help dampen inflammation. Interactions with other transcription factors were shown to repress expression of Il6 and Il1b,^37^ two cytokines often upregulated when innate immunity is activated, or in early stages of adaptive immunity. NRF2 was thus chosen as a potential agent to reduce oxidative damage and inflammation in mouse models of retinitis pigmentosa.^24,25^ The success of this treatment in retinitis pigmentosa suggested it might likewise relieve oxidative stress and inflammation in other diseases, such as dry AMD.

Several models of dry AMD exist, each with their own limitations,^38^ but one of the most commonly used models of dry AMD is rooted in oxidative stress caused by sodium iodate (NaIO_3_).^39,40^ NaIO_3_ is an oxidizing chemical that, when administered through various routes, rapidly causes RPE cell death. RPE loss occurs in patches, with loss occurring preferentially in the central area,^41^ much as AMD occurs in the centrally located macula in humans. Injection of NaIO_3_ is simple, reproducible, and is often used in tests of dry AMD treatments. Here, this model was used to test whether AAV8/Best1-NRF2 could protect against NaIO_3_-induced damage. The expression of NRF2 was limited to the RPE using the BEST1 promoter,^25,42^ as this tissue is affected in AMD and might also be the origin of damage in AMD.^1^ A combination of visual function tests (electroretinography, or ERG), imaging (optical coherence tomography, or OCT), and histological measurements showed that AAV8/Best1-NRF2 effectively counteracted NaIO_3_-induced RPE and photoreceptor cell death. As well, it preserved rod- and cone-based visual function in rat models, and rod-based visual function in mouse models.

## Results

### Characterization of NaIO_3_ Induced Effects in Rodent Models

An NaIO_3_ IP dose of 75 mg/kg was selected for both rats and mice guided by prior literature and preliminary experiments.^43^ Ocular tissues were assayed for RPE loss, photoreceptor cell loss, retinal thinning, and visual function.

**Figure 1:**
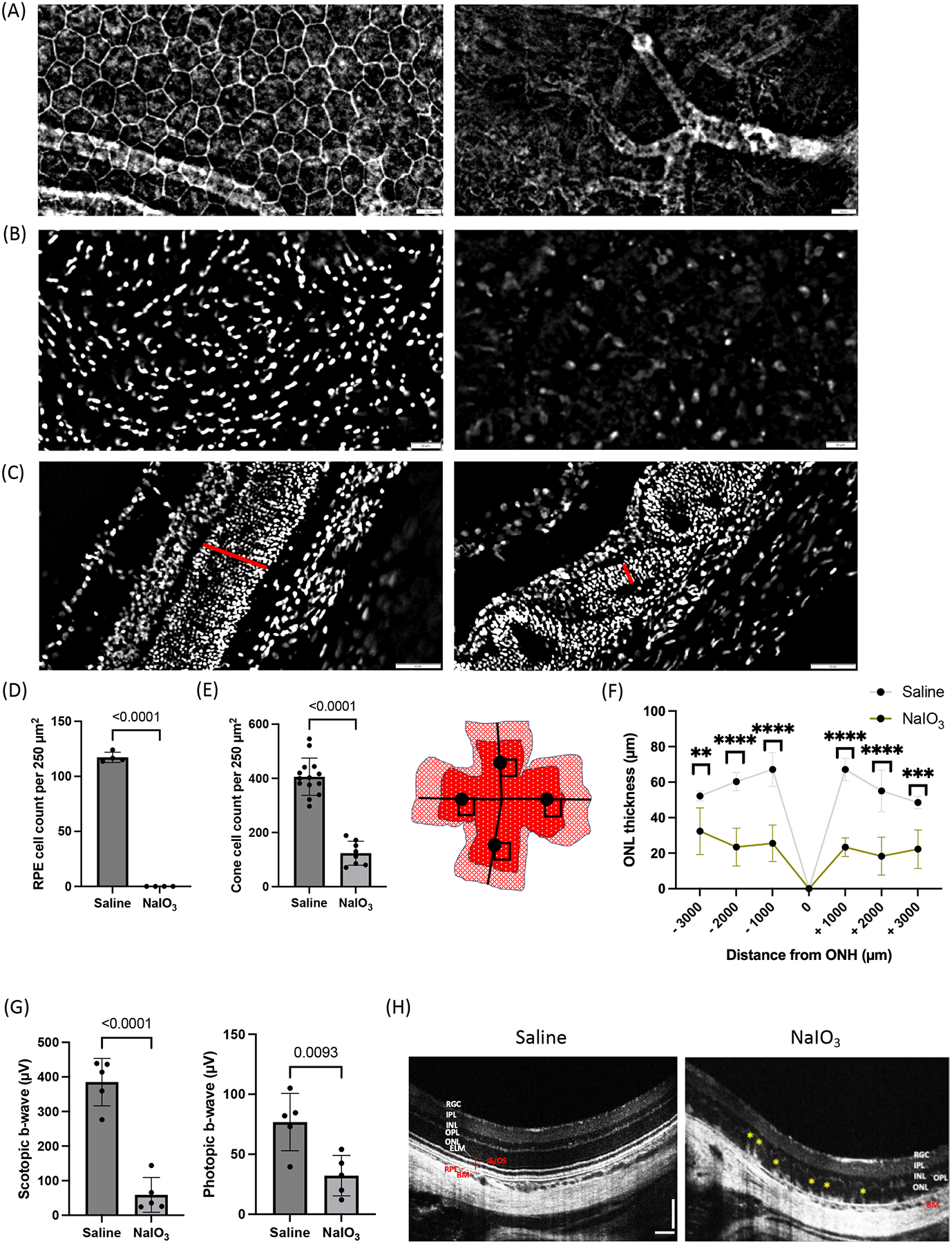
Assessment of RPE and retinal histology after IP injection of NaIO_3_ in rats. Sprague-Dawley rats were IP injected with saline or NaIO_3_ at 6–8 weeks of age. Tissue was harvested 4–5 weeks post IP injection. (A) Representative RPE flatmounts (central region) from a saline (left) or NaIO_3_ (right) IP-injected rat. Flatmounts were stained with phalloidin (white). Scale bar is 20 microns. (B) Representative retinal flatmounts (central region) from a saline (left) or NaIO_3_ (right) IP-injected rat. Flatmounts were stained with an antibody to the cone marker, CAR (white). Scale bar is 20 microns. (C) Representative eyecup sections (mid-peripheral region) from a saline (left) or NaIO_3_ (right) IP-injected rat. Sections were stained with DAPI (white). Red bar indicates the ONL. Scale bar is 50 microns. (D) Quantification of the number of RPE cells remaining (n=4 eyes per group, mean ± SD, p<0.0001; unpaired t-test). (E) Left: Quantification of the number of cones remaining (n=14 saline and n=8 NaIO_3_ eyes, mean ± SD, p<0.0001; unpaired t-test). Right: A diagram of the typical area of the flatmount in which RPE cells or cones die from NaIO_3_ exposure. Dark red (~75% of total) central area denotes dying cells. Black circles represent the midpoint of each line drawn by the investigator for quantification. Boxes are placed with the top left corner at each midpoint. RPE cells or cones are counted in each of the four boxes and the median value is plotted. See Methods for more details. (F) ONL thickness spider plot for eyecup cryosections (n=4–5 per group, mean ± SD, ****p<0.0001, ***p=0.0002, **p=0.008 two-way ANOVA with Šídák’s multiple comparison test). (G) Scotopic ERG b-wave amplitudes (at a 0.1 cd.s/m^2^ flash stimulus, left) and photopic ERG b-wave amplitudes (at a 10 cd.s/m^2^ flash stimulus, right) for rats injected with saline or NaIO_3_ were collected 3–5 weeks post IP injection (n=5 animals per group, mean ± SD, p<0.0001 for scotopic and p=0.009 for photopic; unpaired t-test for each plot). (H) Representative OCT images for a saline IP-injected rat (top panel) or an NaIO_3_ IP-injected rat (bottom panel) at 4 weeks post IP injection. Major retinal layers are annotated as follows: GCL – ganglion cell layer; IPL – inner plexiform layer; INL – inner nuclear layer; OPL – outer plexiform layer; ONL – outer nuclear layer; ECM – external limiting membrane; IS/OS – inner and outer segments of photoreceptors; RPE – retinal pigment epithelium; BM – Bruch’s membrane. Rosettes are highlighted with yellow asterisks. Scale bar is 100 microns.

To determine RPE cell phenotypes following NaIO_3_ or saline IP injections, sclera/choroid/RPE flatmounts were stained with phalloidin.^44^ Phalloidin binds to F-actin, effectively outlining RPE cell morphologies in the RPE monolayer.^44^ RPE flatmounts from saline injected animals showed the characteristic hexagonal, honeycomb-like morphology of healthy RPE cells, whereas NaIO_3_ administration reproducibly resulted in loss of phalloidin signal in the central ~75% of each flatmount in rats and mice (Figure 1A and Figure S1A). Peripheral RPE cells were consistently observed to survive in all flatmounts. Quantification of RPE cells remaining in mid-peripheral areas of each flatmount (calculated as 50% of the distance between the optic nerve and outer edges of each flatmount, see Methods) is shown in Figure 1D and Figure S1D. Loss of phalloidin staining in the central areas of RPE flatmounts, as well as loss of an RPE marker protein, Rpe65, in central areas of eyecup cross sections prepared from rats administered saline or NaIO_3_ (see Methods, Figure S2A–B), suggest that RPE cells in the central regions died upon NaIO_3_ exposure.

To determine the effects of NaIO_3_ IP injections on cones, retinal flatmounts were isolated from the same eyes used for RPE flatmounts and were subjected to immunofluorescence staining for the cone-specific protein, cone arrestin (CAR). CAR is localized throughout the cones, including within cone outer segments,^45^ enabling surviving cones to be recognized as dots on retinal flatmounts. After imaging entire retinal flatmounts, automated image processing algorithms were applied to count the number of cone signals in mid-peripheral regions of the flatmount. As with the RPE, cones in the most peripheral regions of retinal flatmounts typically survived. However, cones located in the central ~75% of each flatmount lost CAR staining following NaIO_3_ injection, indicative of degeneration. The central 75% included “mid-peripheral” cones that were used for quantification of cones located halfway between the optic nerve and outer edges of the flatmount (see Methods). Quantification showed 4-fold fewer mid-peripheral CAR+ cones in rats and mice injected IP with NaIO_3_ compared to saline injected controls (Figure 1B/E and Figure S1B/E).

To determine photoreceptor survival post NaIO_3_ injection, the thickness of the outer nuclear layer (ONL) was measured at fixed distances from the optic nerve in eyecup sections (see Methods). The ONL comprises primarily rods, as they are >90% of photoreceptors in this layer.^46^ The average ONL thickness was significantly lower for NaIO_3_-injected rats and mice, relative to saline-injected controls (Figure 1C/F and Figure S1C/F). In addition to a reduction in thickness, rosettes, or folds which indicate retinal pathology, were observed in the tissue from animals injected with NaIO_3_.

Visual function analyses were also carried out. ERG is used to evaluate the overall physiological status of an animal’s vision^47^ and was evaluated here at baseline, just before NaIO_3_ injection at ~6 weeks of age, and at later time points. ERG was used to measure low-light vision (scotopic b-wave, rod function) as well as brighter light vision (photopic b-wave, cone function). ERG revealed a loss of visual function in NaIO_3_-injected rats (assayed 3–5 weeks post IP) and mice (assayed 3–4 weeks post IP) relative to saline controls (Figure 1G and Figure S1G).

Images of ocular structures were also collected using OCT.^48^ In contrast to saline-injected control animals, NaIO_3_-injected rats and mice showed a loss of overall retinal structure, ONL thinning, and rosettes (Figure 1H and Figure S1H). OCT imaging revealed marked retinal thinning in mice compared to rats at the same dose of NaIO_3_ and at a similar timepoint post IP injection, suggesting mice may be inherently more sensitive to NaIO_3_ damage compared to rats.

### AAV8/Best1-NRF2-Mediated Effects on Retinal Structure and Cells

**Figure 2:**
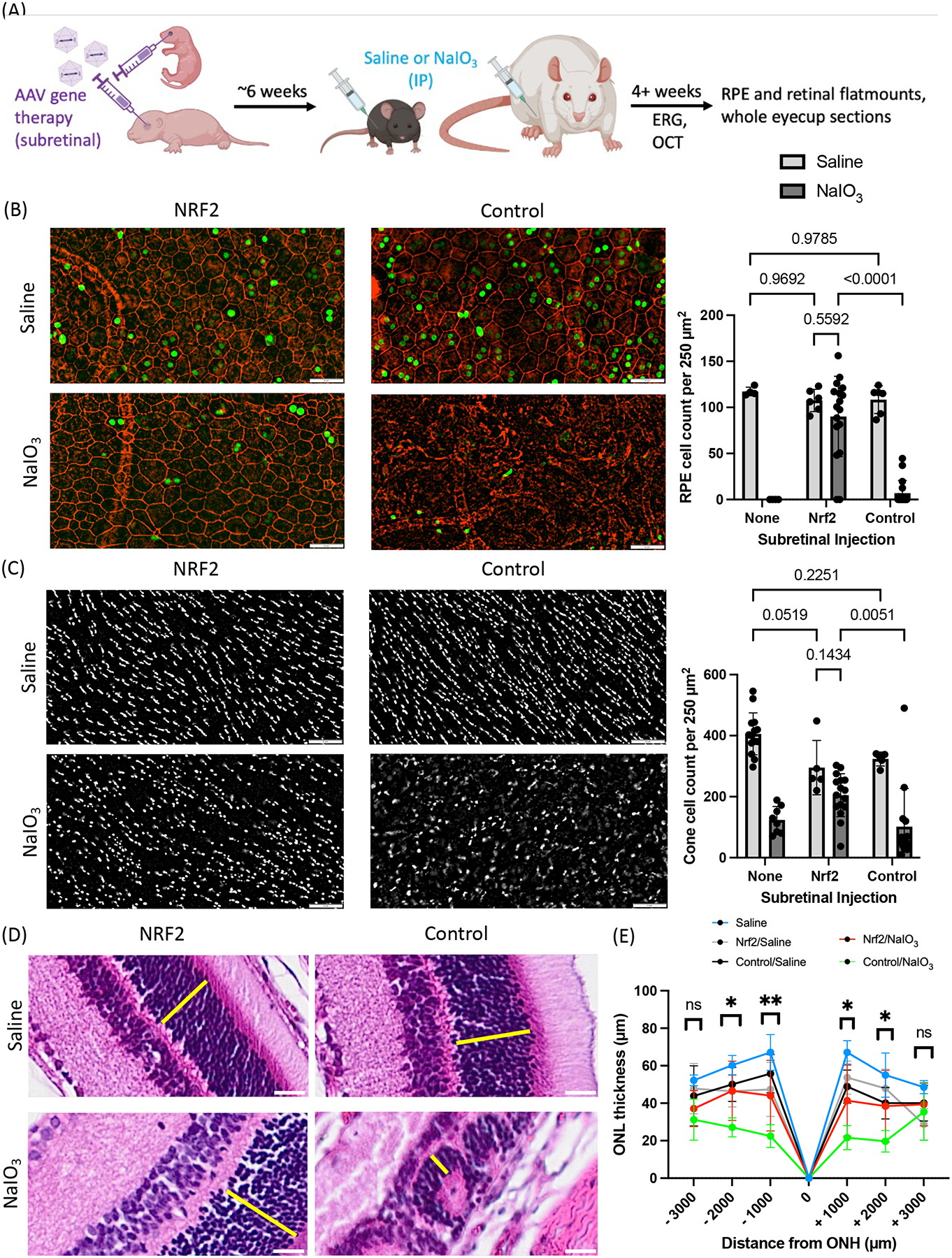
Assessment of AAV8/Best1-NRF2 on RPE and retinal histology in rats. For statistical analysis, all plots below use a two-way ANOVA with Šídák’s multiple comparison test. Figure 1 data serve as “None” controls in this figure. (A) General protocol used to assess efficacy of AAV-mediated NRF2 gene transfer in prevention of NaIO_3_-induced damage. Unless otherwise noted, rats or mice were injected at birth with AAV8/Best1-NRF2 + AAV8/RedO-H2B-GFP in one eye or AAV8/Best1–6xSTOP-mutGFP control vector + AAV8/RedO-H2B-GFP in the contralateral eye and IP injected with saline or NaIO_3_ at 6–8 weeks of age. Ocular tissues were harvested for histology at 4 or more weeks post IP injection. ERG and OCT measurements were collected at one or more timepoints just before IP injection up until just before tissue harvests. (B) Rats were subretinally injected at birth with 2e9 vg AAV8/Best1-NRF2 + 2e9 vg AAV8/RedO-H2B-GFP in one eye or 2e9 vg AAV8/Best1–6xSTOPmutGFP + 2e9 vg AAV8/RedO-H2B-GFP in the other. At P43, rats were IP injected with saline or NaIO_3_. Eyes were harvested at 5–6 weeks post IP injection. Representative RPE flatmounts (central region) stained with phalloidin (red). Green dots in RPE nuclei are H2B-GFP signals, likely from AA8/RedO-H2B-GFP concatemerization with co-injected Best1 promoters to achieve RPE expression of H2B-GFP (see Discussion). Scale bar is 50 microns. Quantification of the number of RPE cells is shown on the right (n=17 NRF2/NaIO_3_ flatmounts, n=17 Control/NaIO_3_ flatmounts, n=6 NRF2/Saline flatmounts, n=6 Control/Saline flatmounts, mean ± SD, p<0.0001 for NRF2/NaIO_3_ vs. Control/NaIO_3_ comparison). Each point in the histogram represents counts from one eye. (C) Rats were injected as described in (B). Representative retinal flatmounts (central region) stained with an antibody to the cone marker, CAR (white). Scale bar is 50 microns. Quantification of the number of cones is shown on the right (n=16 NRF2/NaIO_3_ flatmounts, n=14 Control/NaIO_3_ flatmounts, n=5 NRF2/Saline flatmounts, n=5 Control/Saline eyes, mean ± SD, p=0.005 for NRF2/NaIO_3_ vs. Control/NaIO_3_ comparison). Each point in the histogram represents counts from one eye. (D) Rats were subretinally injected at birth with 2e9 vg AAV8/Best1-NRF2 + 2e7 vg AAV8/RedO-H2B-GFP in one eye or 2e9 vg AAV8/Best1–6xSTOPmutGFP + 2e7 vg AAV8/RedO-H2B-GFP in the other. At 6–6.5 weeks of age, rats were IP injected with saline or NaIO_3_. Eyes were harvested 4–4.5 weeks post IP injection. Representative eyecup sections (mid-peripheral region) stained with hematoxylin & eosin. Yellow bar indicates the ONL. Scale bar is 50 microns. (E) Rats were injected as described in (D). ONL thickness spider plot for DAPI-stained cryosections or H&E-stained paraffin sections (n=4–10 per group, mean ± SD, left to right on plot: *p=0.01, **p=0.003, *p=0.01, *p=0.03 for NRF2/NaIO_3_ vs. Control/NaIO_3_ comparison, two-way ANOVA with Šídák’s multiple comparison test).

An AAV vector expressing human NRF2, driven by an RPE-specific human promoter from the BEST1 gene^42^ and using capsid serotype 8 (AAV8/Best1-NRF2), was previously shown to greatly improve the health of the RPE in a mouse model of retinitis pigmentosa.^25^ This vector was used to treat rodents injected IP with NaIO_3_ in albino Sprague-Dawley rats and pigmented C57BL/6 mice (see Figure 2A for a schematic). As above, IP injections of saline served as a vehicle control.

Viral vectors were injected into the subretinal space of rat or mouse neonatal pups (see Methods for doses of vectors used in each species). As a control for the AAV injection and expression in the RPE, an equal dose of a control virus was delivered to the subretinal space of the contralateral eye. Control vectors used across all experiments were designed such that they did not encode an open reading frame >73 amino acids. The control viruses were one of the following two constructs: (1) an AAV with a BEST1 promoter, two STOP codons in tandem in each reading frame, and an EGFP sequence with 41 point mutations to eliminate any potential start codons (ATG, GTG, and TTG), called AAV8/Best1–6xSTOP-mutGFP, or (2) an AAV with a BEST1 promoter, two STOP codons in tandem in each reading frame, and no EGFP sequence, called AAV8/Best1–6xSTOP.^49^ Unless otherwise noted, another AAV, AAV8/RedO-H2B-GFP, encoding a fusion of histone H2B to GFP (a nuclearly localized fusion protein), was also co-injected into every eye to label the transduced area of each flatmount (see Methods for dosing of GFP vectors). Despite the use of the human red opsin (RedO) cone promoter for expression of H2B-GFP, the RPE was observed to express H2B-GFP. This was not due to a lack of specificity of the RedO promoter, but likely due to concatenation or recombination of the co-injected genomes (see Discussion).

Two litters of rats were injected at birth with the aforementioned vectors. At ~6 weeks of age, they were injected IP with NaIO_3_ or saline. At 4–6 weeks post IP injection, animals were sacrificed and histology was carried out on RPE and retinal flatmounts. Rat RPE flatmounts revealed nearly complete loss of central RPE cells following injection of NaIO_3_ in animals injected with AAV8/Best1–6xSTOP-mutGFP (Figure 2B). In contrast, there was nearly complete preservation of RPE cell numbers after injection of AAV8/Best1-NRF2, when compared with saline-injected control animals. Cones also showed a benefit in AAV8/Best1-NRF2 injected eyes relative to AAV8/Best1–6xSTOP-mutGFP injected eyes, as assessed by CAR labeling of cones on flatmounts (Figure 2C). AAV8/Best1-NRF2 and AAV8/Best1–6xSTOP-mutGFP CAR+ cone numbers were not quite as high as saline-injected controls without AAV infection, but the difference wasn’t statistically significant.

As a measure of rod survival, ONL thickness and retinal morphology were examined in rats. ONL thickness was better preserved after NaIO_3_ injection in AAV8/Best1-NRF2 injected animals compared to AAV8/Best1–6xSTOP-mutGFP injected animals. Hematoxylin & eosin (H&E) staining of sections from paraffin-embedded tissue showed rosettes and disrupted ONL in control virus-injected eyes after NaIO_3_ injection (Figure 2D). Quantification of ONL thickness on these H&E sections and samples processed as cryosections stained with DAPI showed a reduction in control virus-injected eyes relative to AAV8/Best1-NRF2 injected eyes (Figure 2E). The ONL of animals injected with AAV8/Best1-NRF2 or AAV8/Best1–6xSTOP-mutGFP showed modest, but not statistically significant, thinning compared to saline injected controls without AAV infection. Representative DAPI-stained cryosections are provided for reference (Figure S2).

To test whether NaIO_3_ injection triggers inflammation/immune responses in addition to oxidative damage, cryosections from this cohort of rats were also stained for IbaI and GFAP. Iba1 marks infiltrating microglia and macrophages and upregulated GFAP marks cells undergoing gliosis, here Muller glia and astrocytes. ONL infiltration of myeloid cells and induction of gliosis were quite prominent in control/NaIO_3_ samples, but not in NRF2/NaIO_3_ samples or saline controls (Figure S2C–J).

Mice were subjected to a similar protocol as used in rats, with neonatal AAV subretinal injections and young adult IP injections of NaIO_3_. Comparisons were made between eyes injected with AAV8/Best1-NRF2 and control virus. Mice showed similar trends as rats in terms of preservation of RPE cells (Figure S3A/D), cones (Figure S3B/E), and ONL thicknesses (Figure S3C/F) by AAV8/Best1-NRF2 after IP injection of NaIO_3_.

### *In vivo* Measurements of the Effects of AAV8/Best1-NRF2

**Figure 3:**
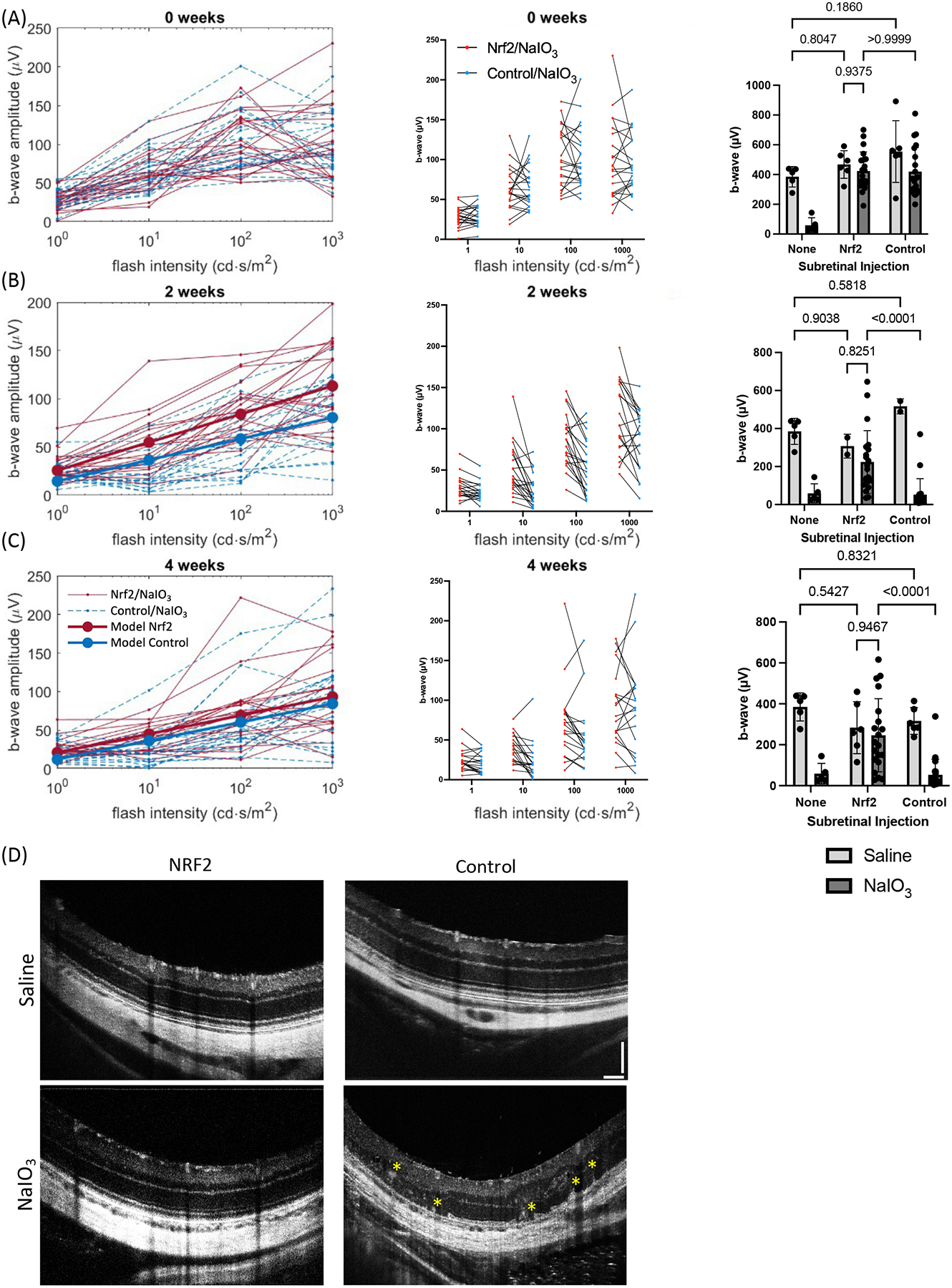
Assessment of AAV8/Best1-NRF2 on retinal structure and visual function in rats. Rats were subretinally injected at birth with 2e9 vg AAV8/Best1-NRF2 + 2e9 vg AAV8/RedO-H2B-GFP in one eye or 2e9 vg AAV8/Best1–6xSTOPmutGFP + 2e9 vg AAV8/RedO-H2B-GFP in the other. At P43, rats were IP injected with saline or NaIO_3_. Eyes were harvested at 5–6 weeks post IP injection. ERG b-wave amplitudes plotted at three timepoints: baseline (before IP injection), 2 weeks post IP injection, and 4 weeks post IP injection of saline or NaIO_3_. For all photopic ERG plots, red lines denote NRF2/NaIO_3_ treated eyes and blue lines denote control/NaIO_3_ treated eyes. For the 2 week and 4 week post IP data (panels B-C), linear mixed-effects models were fit to the data using maximum likelihood with MATLAB’s ‘fitlme’ function and the model fit curves were plotted on top of the data as thicker red/blue lines (denoted Model NRF2 and Model Ctrl in the legend). For scotopic ERG analysis, all plots use a two-way ANOVA with Šídák’s multiple comparison test. Figure 1 data serve as “None” controls in this figure. (A) Left plot: photopic ERG data collected before IP injection with NaIO_3_. Each line represents one eye of a given rat, connected across multiple flash intensities. Middle plot: Same data as in left panel, except lines were drawn to connect the two eyes of a given rat, one of which received an AAV8/Best1-NRF2 injection (red dot) while the other received a control AAV injection (blue dot). Right plot: scotopic ERG data collected before IP injection with saline (n=6 rats) or NaIO_3_ (n=22 rats, mean ± SD). (B) Left/middle plots: same as panel A, except photopic ERG data was collected 2 weeks post IP injection with NaIO_3_. Right: scotopic ERG data collected 2 weeks post IP injection with saline (n=2 rats) or NaIO_3_ (n=21 rats, mean ± SD, p<0.0001 for NRF2/NaIO_3_ vs. Control/NaIO_3_ comparison). (C) Left/middle plots: same as panel A, except photopic ERG data was collected 4 weeks post IP injection with NaIO_3_. Right: scotopic ERG data collected 4 weeks post IP injection with saline (n=6 rats) or NaIO_3_ (n=20 rats, mean ± SD, p<0.0001 for NRF2/NaIO_3_ vs. Control/NaIO_3_ comparison). (D) OCT imaging collected ~4 weeks post IP injection. Rosettes are highlighted with yellow asterisks (scale bar is 100 microns).

In addition to the *post mortem* histological assays described above, the same rats and mice were tested for retinal function and structure using ERG and OCT. Up to 3 timepoints were assayed: (1) immediately before the NaIO_3_ injection to establish baseline visual function/structure, (2) immediately before endpoint tissue harvests, and, in most cases, also (3) ~1–2 weeks post NaIO_3_ injection, as an additional mid-experiment readout of NRF2 effects. ERGs of rats collected at baseline demonstrated healthy photopic and scotopic vision across all eyes of all rats (Figure 3A). At 4 weeks post NaIO_3_ injection, linear mixed-effects models^50,51^ applied across multiple photopic ERG flash intensities showed significant preservation of photopic vision in AAV8/Best1-NRF2-treated eyes relative to control AAV injected eyes (Figure 3B–C, Tables S1–S4). Measurements at 2 weeks also showed preservation, but only 2 saline control animals were assayed at this timepoint. Likewise, preservation of scotopic vision was observed in AAV8/Best1-NRF2-treated rat eyes relative to control AAV injected eyes (Figure 3B–C). Across all timepoints, saline control rats retained healthy scotopic and photopic vision regardless of whether the eye was injected with AAV8/Best1-NRF2 or control AAV (Figure 3A–C). Imaging using OCT showed a retinal rosette phenotype in eyes of animals injected with NaIO_3_ and a control AAV, but not in eyes of animals injected with NaIO_3_ and AAV8/Best1-NRF2 (Figure 3D).

ERG and OCT assays were also conducted on two cohorts of mice. Upon NaIO_3_ injection, scotopic but not photopic vision was retained in eyes injected with AAV8/Best1-NRF2 relative to contralateral control eyes in both mouse cohorts (Figure S4A–B). OCT imaging showed preservation of structure (Figure S4C–E), regardless of whether a control AAV plus GFP tracer AAV was injected in the contralateral eye (Figure S4C–D), or only a GFP tracer AAV was injected in the contralateral eye (Figure S4E). In cases where there was only partial transduction, OCT imaging showed preservation of structure only in the transduced (GFP+) region (Figure S4D).

Four mice from one cohort were held to ~16 months of age prior to one final round of ERGs. The eyes were then processed for cryosectioning and ONL thickness analysis. The ERG data at ~16 months of age looked very similar to the ERG data taken at ~2–3 months of age (e.g., 3–4 weeks post IP injection) for each mouse assayed (Figure S5). Scotopic ERG rescue, observed in 2/4 mice, correlated well with the retention of ONL thickness in those 2 mice (Figure S5A–B). The eyes with preserved scotopic ERGs following AAV8/Best1-NRF2 injection also retained evidence of AAV expression (e.g., AAV8/RedO-H2B-GFP expression was still evident in the sections harvested at this late timepoint). The mice which did not show ONL retention (2/4) also did not show ERG preservation at any timepoint, nor AAV8/RedO-H2B-GFP expression in sections (Figure S5C–D). These data suggest that an assay of structure can predict function, even at very late time points post IP injection.

### Injection of AAV8/Best1-NRF2 in Adult Animals

Delivery of AAV8/Best1-Nrf2 to adult animals was tested to determine if it could provide protection when delivered to fully developed animals, as this would eliminate effects of NRF2 on early postnatal development. Injecting NaIO_3_ into >6 week old rodents, followed by AAV8/Best1-NRF2 subretinal injection, was considered for this purpose. However, as NaIO_3_ injection kills RPE cells as early as 1–3 days post administration,^15^ and expression of AAV-encoded genes can take up to several weeks to reach maximal levels,^52,53^ this order of events was unlikely to provide a meaningful test. Instead, a regimen involving subretinal injection of AAV8/Best1-NRF2 or control AAV into adult eyes, followed by NaIO_3_ or saline IP injection, was adopted. Subretinal injection in mature eyes usually does not result in full transduction, as the inoculum does not fill the subretinal space due to a barrier imposed by RPE-outer segment interactions. This can limit the interpretation of some assays, such as ERG. However, it provides an opportunity to analyze the effects on areas with vector transduction versus those areas without, in the same eye. By co-injecting a GFP virus, the transduced area could be identified for these analyses.

**Figure 4:**
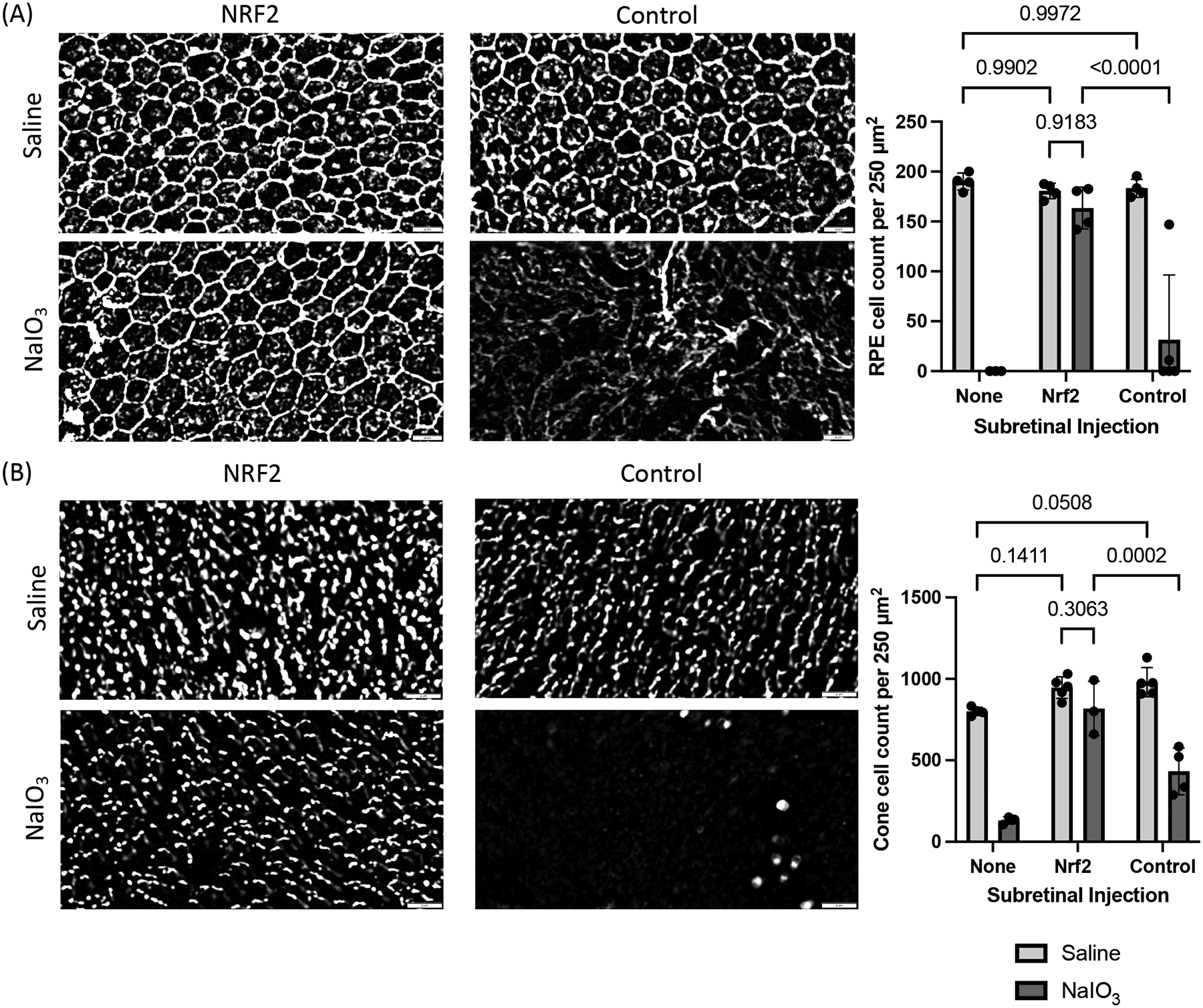
Assessment of AAV8/Best1-NRF2 on RPE and retinal histology in mice subretinally injected as adult animals. C57BL/6J mice were subretinally injected at 6–13 weeks of age with 2e9 vg AAV8/Best1-NRF2 + 2e9 vg AAV8/RedO-H2B-GFP in one eye, or alternatively, 2e9 vg AAV8/Best1–6xSTOP control vector + 2e9 vg AAV8/RedO-H2B-GFP in one eye, and IP injected with saline or NaIO_3_ at 1–2 weeks post subretinal injection. Tissues were harvested/quantified at 4–5 weeks post NAIO_3_ challenge. For statistical analysis, all plots use a two-way ANOVA with Šídák’s multiple comparison test. Figure S1 data serve as “None” controls in this figure. (A) Representative RPE flatmounts (central region) stained with phalloidin (white). Scale bar is 20 microns. Quantification of the number of RPE cells remaining is shown on the right (n=3–5 eyes per bar on graph, mean ± SD, p<0.0001 for NRF2/NaIO_3_ vs. Control/NaIO_3_ comparison). (B) Representative retinal flatmounts (central region) stained with an antibody to CAR (white). Scale bar is 20 microns. Quantification of the number of cones remaining (see Methods) is shown on the right (n=3–5 eyes per bar on graph, mean ± SD, p=0.0002 for NRF2/NaIO_3_ vs. Control/NaIO_3_ comparison).

**Figure 5:**
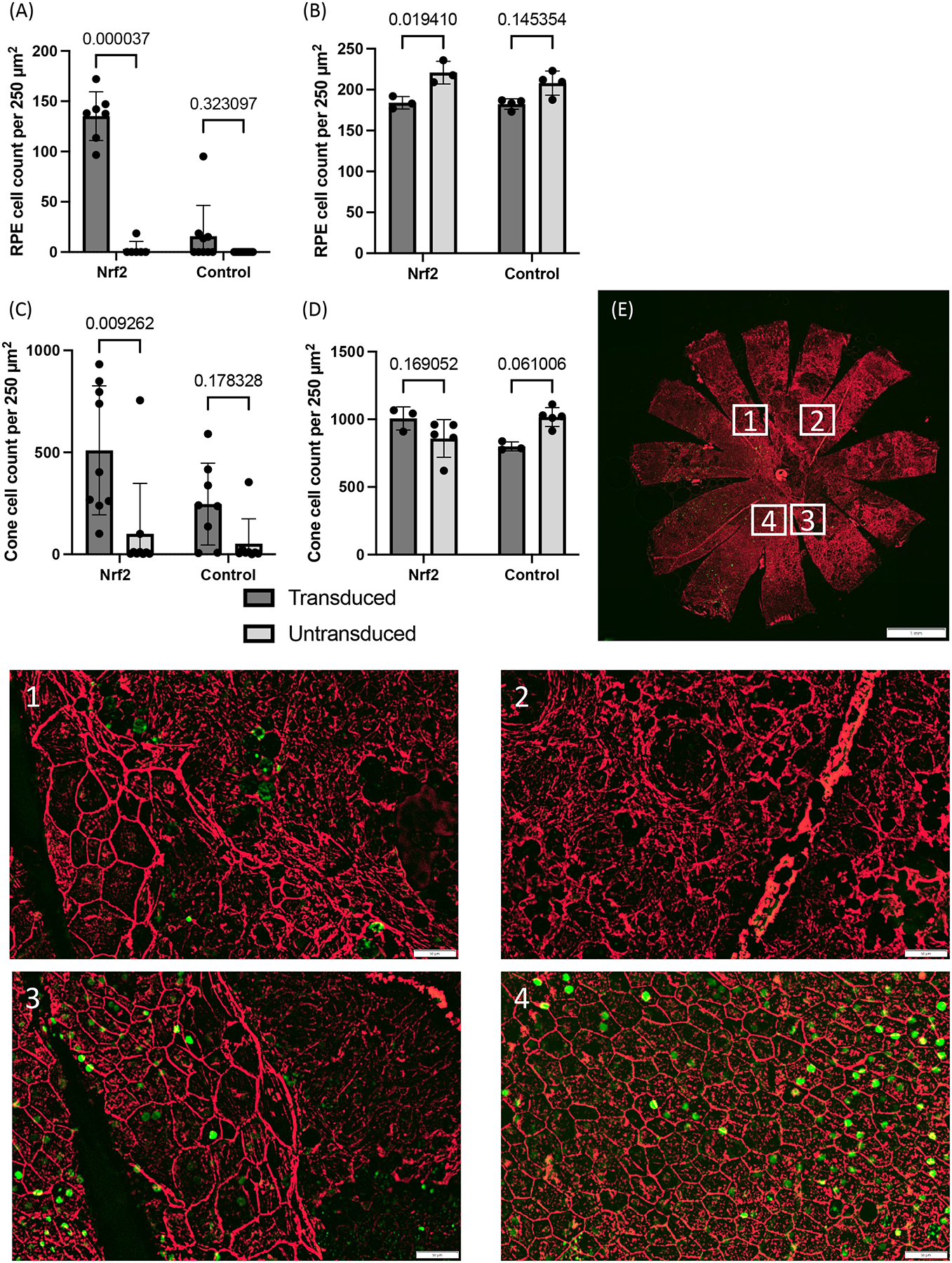
Assessment of local RPE and cone preservation in mice subretinally injected as young adult animals. C57BL/6J mice were subretinally injected at 6–13 weeks of age with 2e9 vg AAV8/Best1-NRF2 + 2e9 vg AAV8/RedO-H2B-GFP in one eye, or alternatively, 2e9 vg AAV8/Best1–6xSTOP control vector + 2e9 vg AAV8/RedO-H2B-GFP in one eye, and IP injected with saline or NaIO_3_ at 1–2 weeks post subretinal injection. Tissues were harvested/quantified at 11–12 weeks post NAIO_3_ challenge, or 4–5 weeks post saline IP injection. Saline control samples are the same as used in Figure 4, re-quantified here for intra-eye comparisons. All panels were analyzed using multiple paired t-tests with Bonferroni-Dunn multiple comparisons test. (A) Quantification of the number of RPE cells remaining in the transduced or untransduced area of each RPE flatmount (n=7 NRF2/NaIO_3_ eyes and n=9 Control/NaIO_3_ eyes, mean ± SD, p<0.0001 for NRF2 comparison). (B) Same as (A), except saline was injected IP (n=3 NRF2/saline eyes and n=4 Control/saline eyes, mean ± SD, p=0.02 for NRF2 comparison). (C) Quantification of the number of cones remaining in the transduced or untransduced area of each retinal flatmount (n=9 NRF2/NaIO_3_ eyes and n=8 Control/NaIO_3_ eyes, mean ± SD, p=0.009 for NRF2 comparison). (D) Same as (C), except saline was injected IP (n=5 NRF2/saline eyes and n=5 Control/saline eyes, mean ± SD). (E) Representative RPE flatmount from a mouse subretinally injected at 6–13 weeks of age with 2e9 vg AAV8/Best1-NRF2 + 2e9 vg AAV8/RedO-H2B-GFP and IP injected with NaIO_3_ at 1–2 weeks post subretinal injection. Scale bar is 1 mm. Phalloidin staining is in red. Within the flatmount, there is an area of live RPE cells (4), areas of transition between live transduced and dead untransduced RPE cells (1, 3), or completely absent RPE cells (2). Area of exposure to AAV8/Best1-NRF2 is evidenced by GFP signal from the co-injected AAV8/RedO-H2B-GFP. Scale bars of subpanel images are all 50 microns.

C57BL/6J mice (age 6–13 weeks old) were subretinally injected with AAV8/Best1-NRF2 + AAV8/RedO-H2B-GFP or AAV8/Best1–6xSTOP + AAV8/RedO-H2B-GFP. IP injections with NaIO_3_ or saline were performed 1–2 weeks post vector injection, and analyses were conducted at 4–5 weeks post vector injection (or later, see Figure 4 and Figure 5 legends). Analysis of RPE flatmounts demonstrated a marked prevention of RPE cell loss in the AAV8/Best1-NRF2 transduced area, in contrast to RPE flatmounts from animals injected with control AAVs, which showed no protection (Figure 4A). Analogously, retinal flatmounts demonstrated increased cone survival in NRF2-transduced retinal patches relative to control virus-transduced retinal patches (Figure 4B). Additional animals were analyzed for RPE/retinal flatmounts for intra-eye, rather than inter-eye, RPE and cone count comparisons (Figure 5). NRF2-transduced areas, identified by AAV8/RedO-H2B-GFP labeling of RPE, retained many more cells than adjacent untransduced areas in the same flatmount (Figure 5A). This rescue effect was not seen in control AAV-transduced eyes (Figure 5A). In RPE flatmounts from control AAV injected animals, the transduced region was deduced from GFP+ patches in surviving RPE cells near the most peripheral edges of the flatmount, where NaIO_3_ was previously noted to show less toxicity to RPE cells. Saline IP injected control mice showed lower RPE counts in AAV transduced areas relative to untransduced areas of the same RPE flatmounts, but this was observed regardless of whether AAV8/Best1-NRF2 or AAV8/Best1–6xSTOP was injected (Figure 5B).

Prevention of cone loss following NaIO_3_ IP injection was higher in NRF2-transduced patches of retinal flatmounts and lower in untransduced areas of the same flatmount (Figure 5C). In control virus injected eyes, transduced areas showed a trend for higher cone counts than untransduced areas of the same flatmounts, but this effect was not statistically significant (Figure 5C). Saline IP injected control animals retained similar cone counts regardless of whether the analyzed region was transduced or untransduced (Figure 5D).

In eyes injected with AAV8/Best1-NRF2 and the AAV8/RedO-H2B-GFP tracer virus and IP injected with NaIO_3_, sharp boundaries delineating RPE survival were seen and were well correlated with transduction (Figure 5E). Higher magnification of areas with or without AAV transduction in the same flatmount revealed the local effects of NRF2 on RPE survival following delivery of AAV8/Best1-NRF2 to adults.

ERG assays were also performed on the mice injected with AAV as adults to determine if visual function was preserved. There was no detectable improvement in visual function in eyes injected with AAV8/Best1-NRF2 or control AAV relative to contralateral uninjected control eyes (Figure S6). Transduction is usually 30–50% of the retina when subretinal injections are performed in adult animals. The lack of detectable rescue effects in the ERG may be attributable to an insufficient number of infected cells.

### Evaluation of NRF2 Pathway Activation

**Figure 6:**
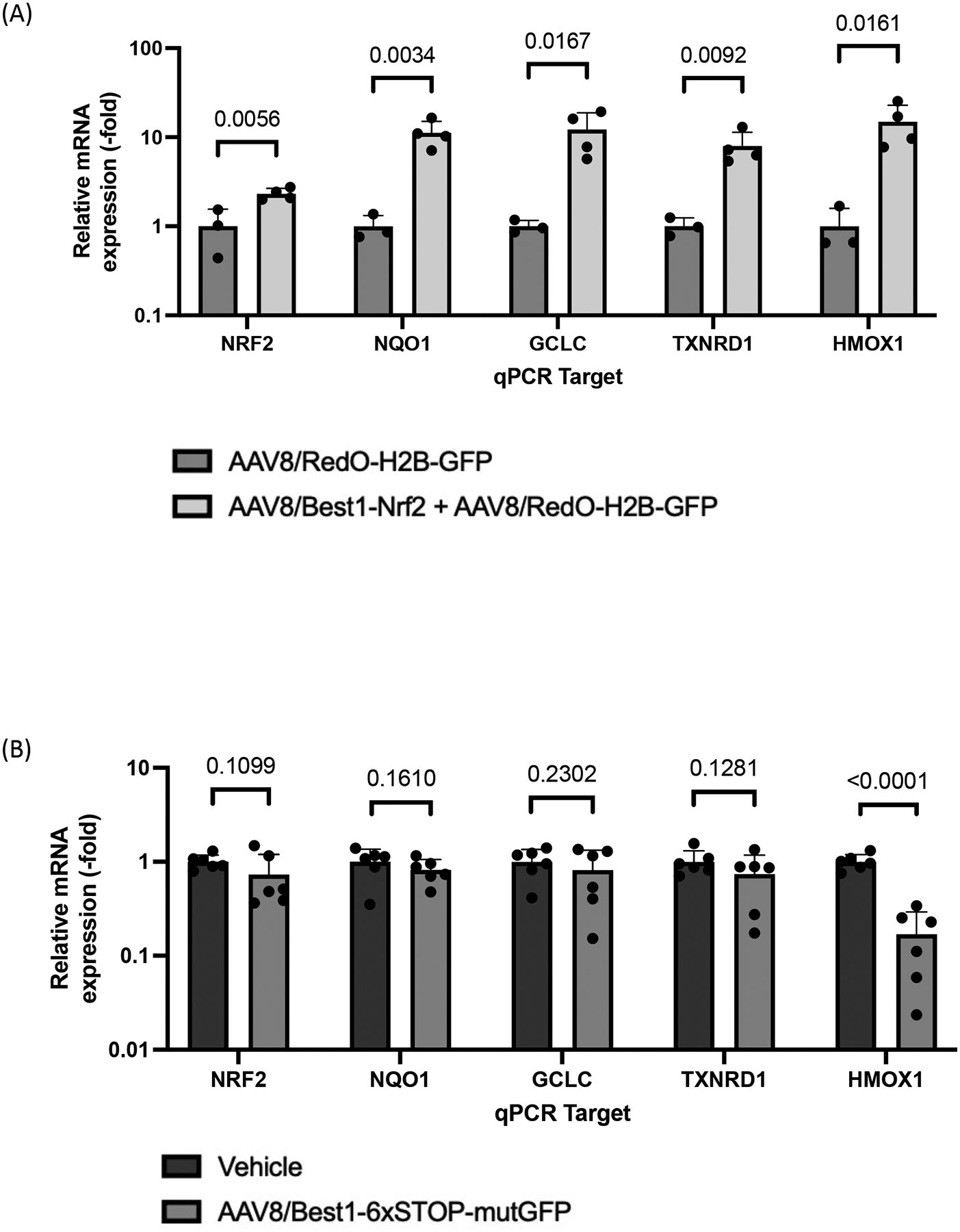
Assessment of NRF2 target gene activation. C57BL/6N mice were injected subretinally at birth with the AAVs/doses indicated below. Eyes were harvested at 7–10 weeks of age for RPE RNA extraction and qPCR quantification of NRF2 transcripts or representative NRF2 target gene transcripts. (A) RPE RNA quantification from mice injected with 4e8 vg AAV8/Best1-NRF2 + 1e7 vg AAV8/RedO-H2B-GFP in one eye or 1e7 vg AAV8/RedO-H2B-GFP in the other eye (n=3–4 eyes per group/target gene assessed, mean ± SD). P-values for t-tests (unpaired, equal variances, 1 tail) are shown. A sign test for the entire group was also run to give a p-value of 0.03 for the group. (B) RPE RNA quantification from mice injected with 4e8 vg AAV8/Best1–6xSTOP-mutGFP control vector in one eye or PBS vehicle in the other eye (n=6 eyes per group/target gene assessed, mean ± SD). P-values for t-tests (unpaired, equal variances, 1 tail) are shown.

To determine if overexpression of NRF2 resulted in activation of its canonical target genes, RNA assays were conducted. One set of neonatal mice was injected with AAV8/Best1-NRF2 + AAV8/RedO-H2B-GFP or only AAV8/RedO-H2B-GFP in contralateral eyes. Another set was injected with AAV8/Best1–6xSTOP-mutGFP control vector or PBS as a vehicle control in contralateral eyes. RPE RNA was extracted from dissected eyes and quantitative PCR (qPCR) was performed to assess expression levels of NRF2, plus four canonical NRF2 target genes: NQO1, GCLC, TXNRD1, and HMOX1 (Figure 6A).^54^ Increased levels of NRF2 RNA (2.3-fold) and that of each target gene (NQO1: 11.2-fold, GCLC: 12.2-fold, TXNRD1: 8.0-fold, and HMOX1: 14.9-fold) were seen in NRF2-transduced RPE relative to control-injected RPE. In addition to analysis of NRF2 target RNAs in eyes injected with AAV8/Best1-NRF2 and AAV8/Best1–6xSTOP-mutGFP, an analysis was done on these target RNAs in control virus injected eyes relative to vehicle injected eyes (Figure 6B). Surprisingly, one NRF2 target gene, HMOX1, was downregulated in control injected RPE relative to vehicle injected RPE (0.17-fold, Figure 6B). The other target genes, and NRF2 itself, were neither upregulated nor downregulated relative to vehicle control RPEs in these control animals (Figure 6B).

### Long-term Tolerability of AAV8/Best1-NRF2

In addition to testing the efficacy of AAV8/Best1-NRF2 in preserving retinal structure and function when administered to rats or mice prior to NaIO_3_ injection, the safety, or “tolerability”, of this potential gene therapy was assessed long-term following injection into otherwise healthy eyes.

**Figure 7:**
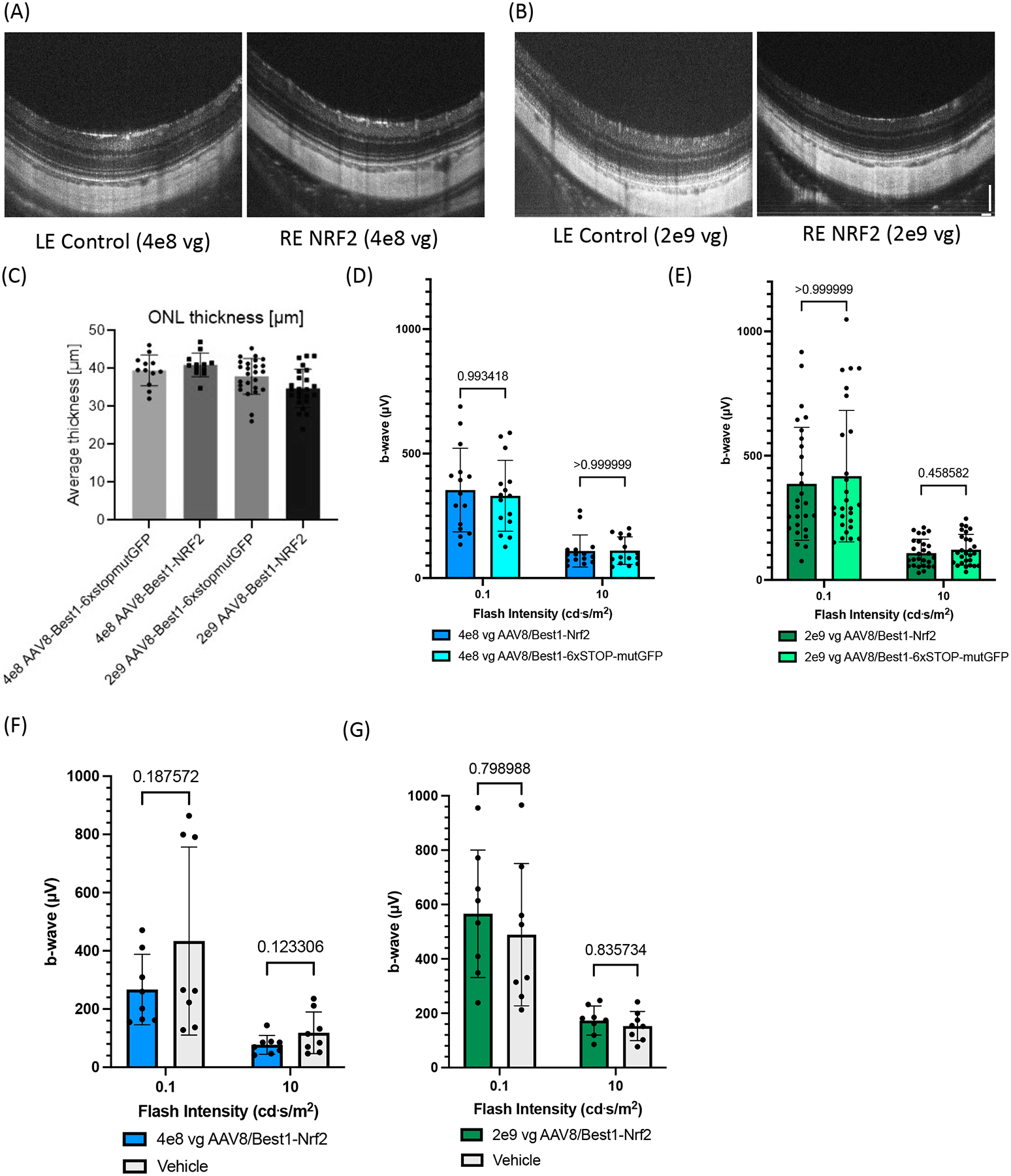
Long-term in-life tolerability assessments for AAV8/Best1-NRF2 in rats. Rats were injected subretinally at birth with AAV8/Best1-NRF2 or AAV8/Best1–6xSTOPmutGFP in contralateral eyes at a dose of 4e8 vg (panels A, C, D) or 2e9 vg (panels B, C, E). Alternatively, rats were injected subretinally at birth with AAV8/Best1-NRF2 or PBS vehicle control in contralateral eyes using an AAV dose of 4e8 vg (panel F) or 2e9 vg (panel G). OCT images were collected at 9–10 months of age and representative images are shown. ERGs were collected at 10–11 months of age and b-wave amplitudes are shown. “Scotopic” ERG used a flash stimulus of 0.1 cd.s/m^2^, while “photopic” ERG used a flash stimulus of 10 cd.s/m^2^. ERG panels were analyzed using multiple paired t-tests with Bonferroni-Dunn multiple comparisons test. (A) Representative OCT images (n=12 rats, scale bar is 100 microns). (B) Representative OCT images (n=25 rats, scale bar is 100 microns). (C) Quantification of ONL thickness from OCT images. (D) Scotopic and photopic ERG b-wave amplitudes (n=15 rats, mean ± SD). (E) Scotopic and photopic ERG b-wave amplitudes (n=27 rats, mean ± SD). (F) Scotopic and photopic ERG b-wave amplitudes (n=8 rats, mean ± SD). (G) Scotopic and photopic ERG b-wave amplitudes (n=8 rats, mean ± SD).

Two cohorts of rats were injected neonatally with AAV, either at a 4e8 vg or 2e9 vg dose per eye. AAV8/Best1-NRF2 was injected in one eye and AAV8/Best1–6xSTOPmutGFP control vector was injected in the contralateral eye. These rats were then aged to ~1 year. OCT measurements were performed on these animals at 9–10 months of age. AAV8/Best1-NRF2 was well tolerated up to a year until terminal necropsy and there were no NRF2-related adverse findings seen in these eyes (Figure 7A–B). Localized disruptions of the retinal structure were occasionally observed. However, these were attributed to trauma associated with the subretinal injection procedure (examples of these disruptions are shown and quantified in Figure S7 and Table S5). From these same OCT images, measurements were made to calculate ONL thicknesses and other metrics representative of retinal health. These included inner nuclear layer (INL) thickness, ONL:INL ratios, and inner limiting membrane to ellipsoid zone, or ILM-EZ thickness. ONL, INL, and ILM-EZ thickness measurements, as well as ONL:INL ratios, were comparable across all doses and AAVs injected (Figures 7C, Figure S8 and Table S6). In general, OCTs revealed thinner retinas relative to images from younger rats, regardless of the treatment and dose. This is compatible with reports indicating that Sprague-Dawley rats have an approximately 50% reduction in the ONL by one year of age.^55^ Notably, the ONL thickness measurements of these 9–10 month old rats, which ranged from ~34–41 microns, all exceeded a previously reported mean ONL thickness for wild type Sprague-Dawley rats at 6 months of age (~28 microns) and 1 year of age (~24 microns).^55^ These observations support the general safety and tolerability of the AAVs at 1 year post-injection into healthy animals.^55^

ERG measurements were also performed on these same rats at 10–11 months of age. ERG results did not reveal differences between scotopic or photopic responses in animals injected with all doses and constructs (Figure 7D–E). To determine if AAV-injected eyes were comparable to non AAV-injected healthy eyes at a younger age, another cohort of rats was injected similar to the above (4e8 or 2e9 vg AAV8/Best1-Nrf2 in one eye), but the contralateral eyes were injected with PBS as a vehicle control. ERG measurements at 1–2 months showed no differences in AAV8/Best1-NRF2 injected eyes relative to vehicle controls (Figure 7F–G).

**Figure 8:**
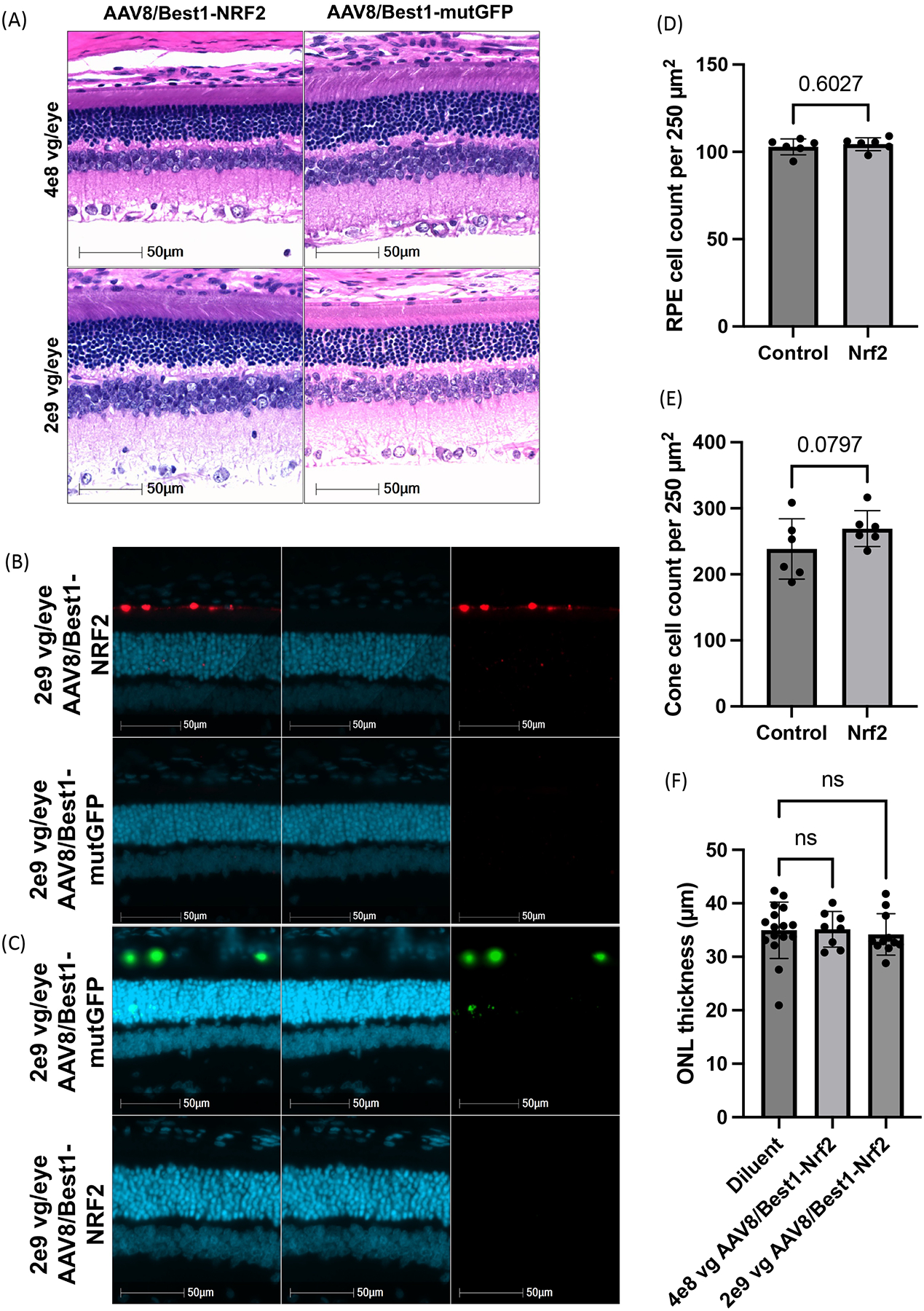
Long-term assessments for AAV8/Best1-NRF2 and assay for presence of viral sequences in rats. Panels A-E: Rats were injected subretinally at birth with AAV8/Best1-NRF2 or AAV8/Best1–6xSTOPmutGFP in contralateral eyes at a dose of 4e8 vg (panel A) or 2e9 vg (panels A-E). OCT images were collected at 9–10 months of age and rats were sacrificed at ~1 year of age (P318-P359). Panel F: Rats were injected subretinally at birth with the indicated dose of AAV8/Best1-NRF2 or a vehicle control in contralateral eyes. Rats were sacrificed at ~11 months (P323-P325) and eyes were processed as H&E stained sections. (A) Representative H&E stained sections from rats injected with 4e8 vg or 2e9 vg of the indicated AAVs at birth and harvested at ~1 year of age (P318-P359). Scale bar is 50 microns. (B) Representative sections from rats injected with 2e9 vg of the indicated AAVs at birth and harvested at ~1 year of age (P318-P359). RNAScope was performed to detect vector-derived NRF2 sequences (red). Sections were also stained with DAPI (blue). Out of 18 NRF2-injected eyes, 15 showed vector derived NRF2 RNAScope signal (83%). Scale bar is 50 microns. (C) Representative sections from rats injected with 2e9 vg of the indicated AAVs at birth and harvested at ~1 year of age (P318-P359). RNAScope was performed to detect vector-derived mutGFP sequences (green). Sections were also stained with DAPI (blue). Out of 18 mutGFP-injected eyes, 15 showed vector derived mutGFP RNAScope signal (83%). Scale bar is 50 microns. (D) Quantification of the number of RPE cells remaining from rats injected with the indicated AAVs at birth and harvested at ~14 months of age (P418, n=6 eyes per group, mean ± SD, paired t-test). (E) Quantification of the number of cones remaining from rats injected with the indicated AAVs at birth and harvested at ~14 months of age (P418, n=6 eyes per group, mean ± SD, paired t-test). (F) Quantification of ONL thickness in midperipheral regions of H&E stained sections from rats injected with the indicated dose of AAV8/Best1-NRF2 or a vehicle control at birth. Rats were harvested at ~11 months of age (P323-P325, n=8–16 eyes per group, mean ± SD, one-way ANOVA with Dunnett’s multiple comparisons correction). A total of 1–2 sections were analyzed per eye and the average ONL thickness was plotted.

A subset of the rats held for OCT and ERG measurements at 9–10 and 10–11 months of age, described above, were sacrificed at ~1 year. The eyes were processed for an additional tolerability assessment via histopathology. Specifically, the eyes were paraffin embedded, sectioned, and stained with hematoxylin & eosin (H&E). A histopathological assessment of the H&E-stained sections confirmed that the AAV8/Best1-NRF2 treatment did not raise any safety concerns at either dose level (Figure 8A). Separate sections were used for RNAScope assays to determine if vector genomes were still present. The probes used were capable of detecting vector genomes, as well as virally encoded RNA, but could not distinguish between them. Out of 18 NRF2-injected eyes, 15 eyes (83%) showed detectable human NRF2 sequence labeling (Figure 8B). RNAScope assay of the mutated GFP sequence in the control vector showed an identical detection rate (15 out of 18 eyes, 83%, Figure 8C). In each case, the probes showed signal only in the cognate infected eyes, i.e. there was no evidence of virus spread from one eye to the contralateral eye, or detection of endogenous sequences. Another subset of these rats (from the 2e9 vg cohort) was sacrificed at ~14 months of age. These eyes were dissected to obtain RPE and retinal flatmounts for additional analyses of RPE and cone survival. RPE and cone cell counts were within the range of normal and comparable between AAV8/Best1-NRF2 and control AAV-infected eyes (Figure 8D–E). Additional rat cohorts injected with vehicle (PBS) in one eye and AAV8/Best1-NRF2 (at a 4e8 vg or 2e9 vg dose) in the other eye were aged to ~1 year prior to harvest. These eyes were paraffin embedded, sectioned, and H&E stained. ONL thicknesses were measured from these sections, and all thicknesses were comparable regardless of whether vehicle or AAV8/Best1-NRF2 was injected (Figure 8F).

Effects of AAV8/Best1-NRF2 were also assessed in mice at two doses of AAV8/Best1-NRF2, 8e7 vg/eye and 4e8 vg/eye, and compared to vehicle-injected contralateral eyes (Figure S9). ERG measurements demonstrated no differences in scotopic and photopic conditions between AAV8/Best1-NRF2-injected eyes and vehicle-injected control eyes at 1–2 months of age (Figure S9A–B). OCT measurements were made on additional cohorts of mice at 8–9 months of age. All retinas were within the range of normal and comparable between AAV8/Best1-NRF2 and vehicle injected eyes (Figure S9C-D). ONL thickness was quantified from these OCT images and again demonstrated comparable values between AAV8/Best1-NRF2 or vehicle injected eyes (Figure S9E).

Throughout the study, we used two different C57BL/6 strains: C57BL/6J, abbreviated B6J here, or C57BL/6NCrl, abbreviated B6N here, from Jackson Labs or Charles River Labs, respectively. This could be a concern as B6N mice have been found to harbor the rd8 mutation, which causes retinal degeneration.^56^ To investigate if strain differences could impact the results, a comparison of histological and functional assays was carried out. RPE and retinal flatmounts, eyecup sections, and ERGs were collected from 16–18 week old B6J and B6N mice. All histological metrics were comparable between the strains, although there was a slightly higher number of RPE cells per 250 μm^2^ region in B6N relative to B6J mice. ERG results of the two strains were also comparable. To test whether C57BL/6J mice, when injected with AAV8/Best1-NRF2 as neonates, could also be rescued similarly to C57BL/6N mice following NaIO_3_ injection, a C57BL/6J cohort was injected at birth with AAVs and IP injected with saline or NaIO_3_ at 8–10 weeks of age. OCT imaging performed ~4 weeks post IP injection indicated that the NRF2-injected eyes of animals challenged with NaIO_3_ were within the range of normal, whereas the contralateral control-injected eyes were not rescued. These data collectively indicate that for all quantitative readouts and timepoints employed in this work, the B6J and B6N strains gave comparable results.

## Discussion

### AMD models and efficacy of AAV8/Best1-NRF2

Oxidative stress underlies many age-related diseases,^57^ and the eye is especially susceptible to oxidative damage due to the high oxygen demands of photoreceptors and the high light environment.^21,23^ Our lab previously demonstrated the efficacy of NRF2-expressing AAVs in reducing oxidative stress and promoting RPE and cone survival in 3 mouse models of RP.^24,25^ Hypothesizing that NRF2 might also be effective in the treatment of another blinding disease with oxidative stress, we tested it in both rat and mouse models of NAIO_3_-induced oxidative damage. There is no single model of dry AMD that fully captures the disease characteristics seen clinically. It is particularly problematic that, among mammals, only some primates have a macula. We chose the NaIO_3_ model because it provides a test of the ability of NRF2 to combat both oxidative damage and inflammation. Given its acute destruction of the RPE, it also provided a challenging model for the protective potential of NRF2. Although the oxidative damage induced by NaIO_3_ is well appreciated, it is less well known that it also causes inflammation and complement deposition. Enzbrenner et al. found complement deposition at early timepoints post NaIO_3_ injection, plus induction of chemokines and inflammatory cytokines.^58^ Our IHC staining for Iba1 and GFAP is consistent with these data, and provides additional support for an anti-inflammatory role for NRF2. While there are other AMD models available, they often require long timeframes (e.g., 1–2 years) to develop phenotypes reminiscent of dry AMD. In addition, the phenotypes do not fully recapitulate dry AMD and are not always fully penetrant.^38^ Some models are more complicated technically than a simple IP injection of NaIO_3_, further supporting our choice of the NaIO_3_ model in this study.

There was striking preservation of RPE and photoreceptors in AAV8/Best1-NRF2 transduced eyes of rats and mice post NaIO_3_ challenge. Efficacy was visualized and quantified using RPE counts, cone counts, and ONL thickness measurements. Using ERG and OCT measurements over time, visual function of rods and cones, as well as retinal morphology, were seen to be retained in NRF2-treated eyes in rats. In mice, ERG assays revealed that rod function, but not cone function, was significantly preserved. These disparate outcomes in ERG results for rats versus mice could be due to inherent variability from several sources: the ERG readout itself, the total number of animals assayed, the subretinal injection quality across individual animals, the sensitivity of each species to the NaIO_3_ injection dose, and/or inherent variability in visual retention across albino vs. pigmented rodent species (discussed more below in the context of OCT). The ERG data from rats are consistent with the retention of cones, as assayed by quantification of CAR-positive cells, as well as by maintenance of ONL thickness, which reflects the survival of rods.

When uninfected animals were injected with NaIO_3_ and the ONL thickness measured by OCT, ONL thinning was more obvious in mouse compared to rat, even though the injected dose of NaIO_3_ was the same (75 mg/kg). This might reflect an underlying greater sensitivity to NaIO_3_ injection in mice compared to rats, and/or it could be due to the fact that the mouse strains used (C57BL/6J or C57BL/6N) are pigmented strains compared to the albino rat strain used (Sprague-Dawley).^40^ Pigmented strains are potentially more susceptible to NaIO_3_ induced damage than albino strains, due to a reaction of the melanin pigment in the RPE with NaIO_3_.^59^ It could also be that C57BL/6 mouse strains are in-bred, and Sprague-Dawley rats are outbred. Genetic factors may lead to a more consistent, more severe response to NaIO_3_ injection in mice, relative to a potentially more variable, less severe effect in outbred rat strains.

### Injection of AAV8/Best1-NRF2 in neonatal vs. young adult animals

Subretinal injections at birth were performed rather than adult injections in the first set of experiments due to several factors. First, neonatal injections produce nearly full RPE and cone transduction as the inoculum readily spreads in neonates, minimizing variability in injection coverage. The oxidative insult, NaIO_3_ IP injection, causes panretinal and acute damage, so full RPE and retinal transduction with AAV8/Best1-NRF2 presents an opportunity to achieve full retinal rescue from NaIO_3_ exposure. Second, the eye can better heal after neonatal injections relative to adult injections, minimizing confounding effects of damage introduced by the injection itself. Third, the expression of transgenes by AAV vector genomes is maximized after ~4 weeks of expression, giving the vector time for NRF2 expression prior to injecting NaIO_3_ in adulthood.^52^

However, there are limitations to neonatal delivery of a gene therapy agent. Firstly, gene therapy delivery to neonatal animals does not necessarily predict the efficacy of delivery to adult humans with ongoing disease. We also cannot rule out the effect(s) of NRF2 overexpression during development, as it might have additional therapeutic effects on development. A requirement for NRF2 in development has at least been addressed, as a NRF2 KO mouse shows no developmental defects.^60^ However, there are no NRF2 overexpression models in the retina available to address this concern. To address both potential limitations of neonatal infections, we tested the delivery of AAV8/Best1-NRF2 in adult mice.

We found that delivery of AAV8/Best1-NRF2 to adult mice prevented NaIO_3_-induced damage. Subretinal AAV delivery to these animals, which gives localized transduction as in humans,^61^ provided an opportunity to evaluate effects of NRF2 within the same animal. Rescue was seen to be local, as RPE and cone survival was seen in transduced regions, but not in untransduced regions, of the same eye. AAV8/Best1-NRF2 would be expected to exert cell-autonomous rescue in RPE cells, since NRF2 is a transcription factor, but it was also possible that healthier, transduced RPE cells could offer some protection to nearby untransduced RPE cells. It was difficult to precisely evaluate this possibility, but if there was non-autonomous rescue of RPE cells, it was restricted to very close neighbors of transduced cells. The protection of photoreceptors was, however, non-autonomous as they do not express genes from the BEST1 promoter. Photoreceptors might have benefitted from the better health of NRF2-overexpressing RPE cells, as RPE cells provide critical support to them. Consistent with this possibility, eyes with localized areas of transduction showed that photoreceptors benefit only in areas of RPE transduction. In addition to healthier RPE cells being better able to provide support to photoreceptors, NRF2 may lead to the upregulation and potentially the secretion of novel factors that provide local benefits for photoreceptors. Additional research is ongoing to test this possibility.

Despite the value of injecting AAV8/Best1-NRF2 into fully developed eyes, there are challenges associated with this injection. First, it is difficult to achieve full retinal and RPE infection. RPE microvilli and photoreceptor outer segments are tightly intermingled in the adult subretinal space, limiting the spread of viral vectors injected into this space. As ERG measurements reflect global retinal function, they may be most impacted by the partial infection in adult eyes. Second, adult rodent eyes don’t heal from the subretinal injection as quickly or completely as neonatal rodent eyes, so injection-related trauma may be evident in histology readouts and/or impact vision assays. These issues, coupled with the possibility that NRF2 doesn’t fix all types of damage elicited by NaIO_3_, may have contributed to a lack of ERG rescue seen in adult mice subretinally injected with AAV8/Best1-NRF2. Even though ERG does not provide evidence supporting functional preservation in adult mice, all histological parameters support local structural preservation in mice injected as adults.

In clinical trials, functional preservation has not been demonstrated in human subjects treated with FDA-approved drugs for dry AMD.^62^ These interventions include two FDA-approved complement inhibitors as well as nutritional supplements (AREDS and AREDS2 formulations).^63^ Common clinical endpoints in dry AMD trials are (1) slowing the rate of lesion growth from the periphery to the central macula, or (2) slowing the enlargement of the area of lesions. These were the endpoints used for the current drugs. Functional analyses are usually considered as secondary endpoints. They are not required for FDA approval due to the difficulty of retaining subjects for functional assessments over sufficiently long periods of time.^62^ As AAV8/Best1-NRF2 therapy provides both structural and functional benefits when injected in rodents, we are hopeful that both structural and functional benefits would similarly be achieved in patients.

We marked transduced areas of RPE and retinal flatmounts in nearly all experiments in the study by co-injecting the AAV8/RedO-H2B-GFP vector with AAV8/Best1-NRF2 or a control AAV into each eye. Despite harboring the human RedO promoter, which is cone-specific,^44,64^ this AAV effectively labeled the transduced area in not just the retina, but also the RPE. RPE labeling was not due to lack of specificity of the RedO promoter, as injection of AAV8/RedO-H2B-GFP alone did not label the RPE.^64^ However, RPE labeling was detected when AAV8/RedO-H2B-GFP was co-injected with an AAV that had a promoter that was active in the RPE. We presume that concatenation or recombination of the two genomes leads to the expression of the H2B-GFP transgene in the RPE. This phenomenon has been reported by Duan et al. (2000) and Coughlin et al. (2025), with a similar interpretation regarding transcriptional cross-talk due to concatenation of co-infecting genomes.^65,66^ Interestingly, the recombination/concatenation did not occur in photoreceptors, as when an RPE promoter driving GFP and a vector with a photoreceptor promoter were co-injected, little or no GFP was seen in the photoreceptors. This may be due to the lower copy number of vectors in the photoreceptors, as when in situ hybridization was used to quantify vector genomes, there was >10 fold fewer genomes in photoreceptors relative to the copy number in the RPE, at a dose comparable to that used here.^67^

### Evidence for AAV8/Best1-NRF2 expression and activity

We verified the expected biological activity of NRF2 as a transcription factor by quantification of RNAs of canonical NRF2 target genes using qPCR. These results are in keeping with our previous study using RNASeq to quantify the effects of AAV8/Best1-NRF2 on RPE gene expression.^25^ We also used IHC to look for NRF2 protein. We could detect it in AAV8/Best1-NRF2 transduced HEK293T cells, which unexpectedly express from the BEST1 promoter. In addition, in the current study, RNAScope probes specific for the AAV-encoded human NRF2 sequence demonstrated the presence of human NRF2 sequences only in AAV8/Best1-NRF2 infected RPE cells harvested from ~1 year old animals. Similarly, probes specific for the control vector showed retention of the control vector at this time point. These analyses showed that the AAV-infected eyes retained vector genomes in the RPE cells after a long period of time.

### Safety of AAV8/Best1-NRF2

AAV8/Best1-NRF2 was well tolerated for ~1 year, with no adverse findings detected across multiple assessments. These assessments included evaluation of retinal function via ERG, histopathological analysis, quantification of retinal structure using OCT, and post-mortem quantification of RPE and cone numbers in flatmounts. Given that the BEST1 promoter and NRF2 cDNA used here were derived from humans, similarly beneficial levels might be predicted if applied clinically. Our study of long-term effects of AAV control vectors with no transgene, or encoding NRF2, also provided data regarding the safety of AAV itself. AAV can cause toxicity, including within the eye, as we have documented for AAV vectors that express in the RPE in mice.^44^ Irrespective of whether a 4e8 vg or 2e9 vg dose of AAV8/Best1-NRF2 or the control AAV, or a PBS (vehicle) control was subretinally injected, ONL thicknesses remained comparable and within a healthy range across all conditions by ~1 year post injection.^55^ For translation to humans, future studies would benefit from extending the dose range to look for any evidence of dose-related safety/tolerability issues.

### Clinical applications of AAV8/Best1-NRF2

AMD and retinitis pigmentosa damage tend to occur in patches, which grow over time.^68^ Delivery of NRF2 to healthier areas adjacent to such patches, or delivered more broadly earlier, before irreversible damage occurs, might slow or prevent loss of RPE cells, much as it prevented NaIO_3_-induced damage. We do not anticipate that AAV8/Best1-NRF2 will lead to the recovery of irreversibly damaged RPE or photoreceptors.

In addition to dry AMD and retinitis pigmentosa, delivery of NRF2 to treat other ocular diseases might be beneficial, as we and others have shown for retinal ganglion cell injury.^24,69^ Other diseases of the CNS similarly have shown some benefit from NRF2 activity.^34,70^ Given these data, and the efficacy and general safety results demonstrated here, delivery of NRF2 to other organ systems, where oxidative damage and inflammation occur, may also prove to be beneficial.

## Materials and Methods

### Animals

Animals were handled according to protocols approved by the Institutional Animal Care and Use Committee (IACUC) of Harvard University (IACUC mouse protocol #1695, IACUC rat protocol #3495).

Unless otherwise noted, C57BL/6NCrl (Strain #027) untimed or timed pregnant female mice were purchased from Charles River Laboratories to obtain litters for neonatal mouse subretinal injections. Adult C57BL/6J (Strain #000664) males and females were purchased from Jackson Laboratories or bred in-house to obtain adults needed for mouse NaIO_3_ model establishment and all adult subretinal injection experiments. Untimed pregnant Sprague-Dawley female rats were purchased from Charles River Laboratories (Strain #001) and/or bred in house. Animals were bred and maintained at Harvard Medical School (HMS) on a 12-hour alternating light/dark cycle. The breakdown of male and female animals used for experiments in this study is provided in Tables S7–S8.

### AAV Vector Design and Preparation

AAV8/Best1-NRF2 was prepared by Spark Therapeutics and used for all experiments performed in this work. This vector was produced by triple transfection of HEK293 cells, and concentrated by cesium chloride gradient purification.^71^

Control AAVs (AAV8/Best1–6xSTOP and AAV8/Best1–6xSTOPmutGFP) as well as the tracer GFP AAV (AAV8/RedO-H2B-GFP) were prepared at HMS for all experiments performed in this work. AAV8 vectors were packaged in HEK293T cells using standard transient transfection protocols and concentrated via iodixanol gradient ultracentrifugation as previously described.^44^ Titers of AAV batches prepared at HMS, as well as the AAV8/Best1-NRF2 vector stock from Spark Therapeutics, were determined by running two-fold dilutions of stocks of unknown titers on a protein gel alongside a dilution series of a stock of AAV of known titer derived by PCR of genomic sequences.

### AAV Subretinal Injections

AAV was delivered by subretinal injection into P0-P2 C57BL/6NCrl pups, P0-P2 Sprague-Dawley rat pups, or 6–13 week old C57BL/6J adult mice using hand-pulled glass needles attached to an Eppendorf FemtoJet Express Microinjector (Eppendorf 5248000261, discontinued) as previously described for pups.^72^ PBS was used to dilute AAV solutions to the correct concentrations used for injections. AAV solutions were prepared with 0.01% wt/vol Fast Green FCF dye to visualize the injection bleb. Where noted, a vehicle control consisted only of 0.01% wt/vol Fast Green FCF dye in PBS injected subretinally. Animals were administered the analgesic buprenorphine (0.05–0.1 mg/kg, Par Pharmaceutical, NDC 42023–179-01) subcutaneously by a 31G insulin syringe (BD cat #SY8290328291) just prior to the subretinal injection, and a drop of Proparacaine Hydrochloride ophthalmic solution, USP 0.5% (Akorn, NDC 17478–263-12) into each eye post subretinal injection. For adult rodent subretinal injections, a small amount of Terramycin ointment (Zoetis, NADA #8–763) was additionally added to each eye post subretinal injection as a lubricant and antibiotic.

#### Mouse/Rat Pup Injections:

Pups were briefly anesthetized by cryoanesthesia on a paper towel on ice. The palpebral fissure was opened with a 30G needle. The eye was gently popped out of the socket with blunt forceps, and AAV diluted in PBS was injected into the subretinal space. Both left and right eyes were used, with the contralateral eye often serving as a control for the experimental condition (e.g., left eye would receive AAV8/Best1-NRF2, right eye would receive an equivalent dose of AAV8/Best1–6xSTOP-mutGFP or AAV8/Best1–6xSTOP control AAV). To avoid bias in injection coverage, some animals received AAV8/Best1-NRF2 in the left eye and the remaining received it in the right eye. Where noted, AAV8/RedO-H2B-GFP was co-injected as an injection tracer to demarcate the transduced area of flatmounts.

#### Adult mouse injections:

Adult mice were given an IP administration of ketamine (80 mg/kg) and xylazine (10 mg/kg) diluted in PBS for temporary anesthesia. The eye was held open and a 30G needle was used to create a small hole at the base of the cornea (at the corneal-scleral interface) for a hand-pulled needle to enter through. AAV was then injected into the subretinal space. For each animal, the same AAV solution (AAV8/Best1-NRF2 or AAV8/Best1–6xSTOP control AAV, along with AAV8/RedO-H2B-GFP as an injection tracer) was injected into either the right eye only, or both the left and right eyes. Right eyes were preferred for injections due to potentially better injection coverage compared to the left eye. At 1–2 weeks post subretinal injection, adult mice were administered saline or NaIO_3_ via IP injection. Eyes were harvested for histological analyses between 4–5 or 11–12 weeks post IP injection as noted in the figure legends.

The AAV8/Best1–6xSTOPmutGFP control vector was used for neonatal rat/mouse subretinal injections, whereas the AAV8/Best1–6xSTOP control vector was used for adult mouse subretinal injections. These vectors are considered interchangeable for experimental purposes because saline control animals showed minimal/no RPE toxicity regardless of the control vector injected. For some neonatal mouse experiments, no control vector was injected, and the control eye consisted only of an AAV8/RedO-H2B-GFP injection (as noted in figure legends).

### Typical Subretinal Injection Doses for AAV8 Vectors, Unless Otherwise Noted:

Abbreviations (for dosing descriptions below): vg = vector genomes; NRF2 = AAV8/Best1-NRF2 vector; Control = AAV8/Best1–6xSTOPmutGFP vector (used for all rat/mouse pup injections) or AAV8/Best1–6xSTOP vector (used for adult mouse injections); GFP = AAV8/RedO-H2B-GFP tracer vector

#### Mouse pups:

NRF2/Control vectors were each diluted to 1.6e9 vg/μL prior to injections. GFP vector was diluted to 8e8 vg/μL prior to injections. Approximately ~0.25 μL was injected per eye.

#### Rat pups:

In most experiments, NRF2/Control/GFP vectors were each diluted to 2e9 vg/μL prior to injections. Where noted in figure legends, AAV8/RedO-H2B-GFP was used at a 100-fold lower dose, so was diluted to 2e7 vg/μL prior to injections. Approximately ~1–2 μL AAV was injected per eye.

#### Adult mice:

NRF2/Control/GFP vectors were each diluted to 2e9 vg/μL prior to injections. Approximately ~1–2 μL AAV was injected per eye.

### Preparation of Glass Needles for Subretinal Injections

Needles for subretinal injection were made from Wiretrol II glass capillaries (Drummond #5–000-2005). Capillaries were pulled on a Model P-97 Flaming/Brown Micropipette Puller from Sutter Instrument Co. with the following settings: P (Pressure Setting) = 500, Heat = 645, Pull = 30, Vel. (Velocity) = 45, Time = 50. Needles were sharpened on a Narishige Micro Grinder (#EG-401) with the angle set to 30° and grinding disk speed to ~70. As the eyepiece includes a micrometer, needle tips were adjusted to be ~1 μm thick and sharpened until they had a visible bevel and point.

### Intraperitoneal (IP) Injections

Rats and mice were IP injected with a freshly prepared solution of NaIO_3_ (Sigma, cat # S4007–100G) diluted in sterile 0.9% saline (Fisher Scientific, #Z1376) delivered at a dose of 75 mg/kg. For rat experiments, roughly 25% of rats (n=6) were set aside for vehicle control (saline only) IP injections. For mice injected subretinally as adults, roughly 50% were set aside for saline IP injections due to the uncertainty of infection success. For mice injected subretinally at birth, due to limiting numbers of litters, none were used for saline IP control injections so that they could be entirely allocated to NaIO_3_ injections. Animals injected subretinally at birth were typically aged to 6–8 weeks before performing IP injections unless otherwise specified. For mice subretinally injected as adults, 6–13 week old mice were subretinally injected with AAVs, then IP injected with saline or NaIO_3_ at 1–2 weeks post subretinal injection.

### Optical Coherence Tomography (OCT)

After inducing general anesthesia, 0.5% tropicamide eye drops were used to dilate the rodent’s eyes. Eyes were then lubricated with an ophthalmic gel to avoid corneal desiccation. The examined eye was then positioned for imaging in a Phoenix Micron IV (MICRON Image-Guided OCT2 system). The adjustable holder was rotated/moved to the capture line to view the fundus image and retinal structure. The optic nerve head (ONH) was positioned on the nasal edge of the imaged area and used as a landmark. The scan line was placed from the ONH to the periphery, and each parameter was adjusted to capture more than three OCT images per eye. The images were analyzed using Insight software (Phoenix Research Labs) for retinal layer segmentation and ONL thickness quantification, following established protocols.^73^

Retinal layers were identified in rat tolerability OCT images as follows. The inner nuclear layer was defined as a hyporeflective band between a more highly reflective nerve fiber and ganglion cell layer on one side and a hyperreflective band corresponding to the inner plexiform layer on the other side. The outer nuclear layer was identified as a hyporeflective band between the hyperreflective inner plexiform and a high reflectivity band corresponding to the external limiting membrane. Measurement of the neurosensory retinal layer thickness from the inner limiting layer (ILM) to the ellipsoid zone (EZ) was also performed. To quantify the ILM to EZ thickness, outer nuclear layer (ONL) and inner nuclear layer (INL), captured images were copied to Microsoft PowerPoint software and a distance measurement tool was used to determine the length of the layer of interest in inches. These values were then converted to microns by measuring the scale bar present within each image, dividing 100 μm by the obtained value in inches for the scale bar and then multiplying each thickness measurement by this number to obtain each thickness in μm.

### Measurements of RNA for NRF2 Target Genes

After sacrificing the mice and removing their eyeballs, the cornea, iris epithelium, and lens were gently removed and discarded. The RPE layer was separated from other tissues by digestion with Dispase (Stem Cell Technologies, #07913) for a 45 min incubation at 37°C. This was followed by scraping off the choroid, adapting the protocol introduced by Shen et al.^74^

Total RNA from the RPE layer was then extracted using a Direct-zol RNA MiniPrep with TriReagent kit (Zymo, #R2051-A) according to the manufacturer’s instructions. cDNAs were synthesized using a SuperScript III First-Strand Synthesis System kit (Life Technologies Corporation, #18080051). Quantitative PCR amplifications were performed with AzuraView GreenFast qPCR Blue Mix (Azura Genomics, #AZ-2305). The relative expression values of each gene were determined by normalization to HPRT1 expression for each sample. The normalization method was double delta Ct analysis. Primer sequences were as follows:

Mouse NQO1-forward primer: AGGATGGGAGGTACTCGAATC
Mouse NQO1-reverse primer: TGCTAGAGATGACTCGGAAGG
Mouse GCLC-forward primer: GGGGTGACGAGGTGGAGTA
Mouse GCLC-reverse primer: GTTGGGGTTTGTCCTCTCCC
Mouse TXNRD1-forward primer: GGGTCCTATGACTTCGACCTG
Mouse TXNRD1-reverse primer: AGTCGGTGTGACAAAATCCAAG
Mouse HMOX1-forward primer: AAGCCGAGAATGCTGAGTTCA
Mouse HMOX1-reverse primer: GCCGTGTAGATATGGTACAAGGA
Mouse HPRT1-forward primer: CAGTCCCAGCGTCGTGATTA
Mouse HPRT1-reverse primer: TGGCCTCCCATCTCCTTCAT
*NRF2-forward primer: GGACATGGATTTGATTGACATACTT
*NRF2-reverse primer: AGTTTTTTCTGTTTTTCCAGCTCATA

*Because the NRF2 plasmid used in this study encodes human NRF2, this pair of primers was designed to target the shared part of the mRNA sequence of mouse (isoform 1) and human (isoform 1) NRF2. This pair of primers spans exons to avoid targeting genomic DNA.

### Electroretinography (ERG)

Scotopic and photopic ERGs were collected for dark-adapted rats as previously described.^75^ Ganzfeld ERG was performed using an Espion E3 System (Diagnosys LLC). Data were analyzed using the Espion V6 software. Scotopic and photopic ERGs were collected for rats and mice who were dark adapted for at least 2 hours prior to the start of the experiment. Anesthesia was induced by IP injection of a ketamine/xylazine cocktail (80/10 mg/kg) diluted in sterile PBS. Pupil dilation was achieved by adding one drop of Tropicamide 1% (Bausch + Lomb) solution to each eye. Animals were placed on a 39°C heating pad and the head was stabilized inside of a Ganzfeld bowl illuminator. A platinum reference electrode was inserted subcutaneously in the midline of the anterior scalp and a platinum ground electrode was placed subcutaneously in the dorsal midline of the animal in the area of the lumbar vertebrae. Gold corneal electrodes were then placed onto both eyes along with PBS drops to keep eyes moist during ERG acquisition. Scotopic testing was performed first, at an intensity of 0.1 cd.s/m^2^. Both eyes were measured at the same time. Ten measurements were taken and averaged. The animals were then light adapted for six minutes, followed by photopic testing. This consisted of white flashes applied to elicit ERG responses at multiple, increasingly bright intensities of 1, 10, 100, and 1000 cd.s/m^2^ with a 30 cd.s/m^2^ white light background. Thirty, thirty, five and four measurements were taken at 1, 10, 100, and 1000 cd.s/m^2^, respectively, and averaged. Both eyes were measured at the same time with the electrical impedance balanced. After ERG acquisition, Terramycin (Zoetis) antibiotic ophthalmic ointment was applied to each eye to prevent infection and keep eyes moist until each animal fully recovered from anesthesia.

### Histology

Mice and rats were euthanized by CO_2_ overdose followed by cervical dislocation. Eyes were enucleated and then processed differently depending on the desired readout: (1) the sclera/choroid/RPE layer was collected for RPE flatmounts, (2) the neuroretina layer was collected for retinal flatmounts, or (3) the eyecup (anterior chamber and lens removed, RPE + retina intact) was collected for section-based analysis. All tissues were fixed in 4% paraformaldehyde (Thermo Scientific, Cat #28908) diluted in PBS on a rocking shaker for at least 4 hours at room temperature or overnight at 4°C. After fixation, tissues were washed 3 times in PBS. Flatmount samples were directly stained with primary antibodies diluted in freshly prepared staining buffer (1% Triton X-100, 4% normal donkey serum in PBS). Samples intended for sectioning were instead frozen for processing on a cryostat as described below.

#### RPE flatmounts:

After washing, RPE flatmounts were incubated in phalloidin (1:100 dilution in staining buffer). Alexa Fluor 647 Phalloidin (Invitrogen #A22287), Alexa Fluor 568 Phalloidin (Invitrogen #A12380), or Alexa Fluor 488 Phalloidin (Invitrogen #A12379) were used interchangeably. Phalloidin incubations of 4 hours at room temperature or overnight at 4°C were sufficient for mouse samples, whereas overnight (or 1–3 day) incubations at room temperature were used for rat samples. After staining, samples were washed 3 times in PBS and mounted RPE side down onto coverslips (VWR, 24×50mm No. 1.5, #48393–241) in Fluoromount G mounting solution (Southern Biotech) on Superfrost Plus slides (Fisher Scientific, #22–037-246) prior to imaging.

#### Retinal flatmounts:

After washing, retinal flatmounts were incubated in CAR primary antibody (Millipore Sigma #AB15282, 1:3000 for mouse samples or 1:2000 – 1:3000 for rat samples) diluted in staining buffer. Incubation times for mouse samples were the same as those specified above for RPE samples, and overnight room temperature incubations were sufficient for rat samples. After staining, samples were washed 3 times in PBS, incubated in secondary antibody (Alexa Fluor^®^ 647 AffiniPure^™^ Donkey Anti-Rabbit IgG (H+L), Jackson ImmunoResearch, #711–605-152, 1:750 dilution in PBS) for 2–4 hours at room temperature, washed 3 times in PBS, and then mounted with the ONL side down onto coverslips (VWR, 24×50mm No. 1.5, #48393–241) in Fluoromount G mounting solution (Southern Biotech) on Superfrost Plus slides (Fisher Scientific, #22–037-246) prior to imaging.

#### Eyecup Sections:

After washing, eyecups (i.e. retina and attached RPE/sclera) were allowed to equilibrate in 10% sucrose in PBS until they sank, then they were transferred to a solution of half Optimal Cutting Temperature (OCT) compound and half 30% sucrose for at least 30 minutes at 4°C. Eyecups were frozen in cryomolds (VWR Tissue-Tek Biopsy Cryomolds, 10 mm × 10 mm × 5 mm, #25608–922) and stored at −20°C or −80°C prior to sectioning on a Leica CM 3050S cryostat (Leica Microsystems). Sections were cut at a thickness of 20 μm onto Superfrost Plus slides (Fisher Scientific, #22–037-246) prior to staining with DAPI (Invitrogen) for 15+ minutes. Fluoromount G mounting solution was then added to slides and coverslips were placed prior to imaging, as above.

For Rpe65 staining, sections were cut as described above and then samples were incubated in a staining solution containing the Rpe65 antibody (Abcam #ab231782, 1:250 dilution in staining buffer) and DAPI for 5–6 hours at room temperature. Sections were then washed with PBS and incubated in secondary antibody solution (Alexa Fluor^®^ 488 AffiniPure^™^ Donkey Anti-Rabbit IgG (H+L), Jackson ImmunoResearch, #711–545-152, 1:750 dilution in PBS) for 2 hours at room temperature prior to a final wash with PBS. Fluoromount G mounting solution was then added to slides and coverslips were placed prior to imaging, as above.

For IbaI and GFAP staining, sections were cut as described above and sections were incubated in a staining solution containing either the IbaI antibody (Novus Biologicals #NB100–1028, 1:200 dilution in staining buffer) or the GFAP antibody (Aves Labs, 1:500 dilution in staining buffer) overnight at room temperature in a humidified chamber. Sections were then washed 3x in PBS (5+ min/wash) and incubated in secondary antibody solution containing DAPI for 2–4 hours at room temperature. The following secondary antibody was used for IbaI: Cy3 Affinipure Donkey anti-Goat IgG, Jackson ImmunoResearch, #705–165-147, 1:750 dilution in PBS. The following secondary antibody was used for GFAP: Alexa Fluor^®^ 647 AffiniPure^™^ Donkey Anti-Chicken IgG (H+L), Jackson ImmunoResearch, #703–605-155, 1:750 dilution in PBS. After secondary antibody incubations, samples were washed 3x in PBS and coverslipped prior to imaging.

#### Whole Eye H&E Preparation:

Eyes were harvested from rats (Figure 2D–E) at 4–5 weeks post IP injection. Eyes were immediately placed in 4% paraformaldehyde (with a corneal slit to allow for better fixative penetration) for overnight incubation at 4°C. The next day, samples were delivered to the Schepens Eye Research Institute of Mass Eye and Ear Morphology Core for paraffin embedding, 6 μm sectioning, and H&E staining. A separate set of eyes harvested from NaIO_3_ injected mice (Figure S3F, 12–13 weeks post NaIO_3_ injection, no corneal slit made) was fixed overnight in Davidson’s fixative at 4°C prior to delivering samples to the Schepens Eye Research Institute of Mass Eye and Ear Morphology Core for paraffin embedding, sectioning, and H&E staining. Davidson’s fixative was prepared by mixing 300 mL 95% ethyl alcohol, 200 mL 10% neutral buffered formalin, 100 mL glacial acetic acid, and 300 mL distilled water. Uninjected 7–9 week-old B6J control eyes (fixed with either Davidson’s fixative or 4% paraformaldehyde overnight at 4°C) were also submitted for identical H&E processing by the Morphology Core as healthy controls for the mouse NaIO_3_ samples above.

### H&E Staining

For rat tolerability samples (Figure 8), paraffin blocks were sectioned at 5 μm, baked overnight at 60°C, and stained for H&E on a Leica Autostainer XL. Images were captured on Axioscan 7 digital slide scanner (Zeiss) and reviewed by a board-certified veterinary pathologist.

### RNAScope

Two staining methods were used for the RNAscope assays. Both staining methods were performed on a Leica Bond Rx. An RNAscope LS Multiplex Reagent Kit (ACDbio, #322830) was used. Slides were pretreated with a Bake and Dewax (Leica, #ar9222). Heat induced epitope retrieval was performed with ER2 (EDTA) for 15 minutes at 95°C followed by 15 minutes exposure to ACD protease. Slides were incubated with probes for 120 minutes. Probes Hs-nfe212-o2-c2 (ACDbio, #1836648-c2) and EGFP-O2–027-c3 (ACDbio #1862748-c3) were stained with Opal 690 (Akoya, #op-001006) at a dilution of 1:1000. Images were captured on an Axioscan 7 digital slide scanner (Zeiss).

### Microscopy

All coverslipped flatmounts and sections were imaged on one of the following: (1) an Olympus VS200 Slide Scanner with a UPlan X Apo 10x/0.4 Air objective, (2) a Nikon Ti inverted microscope with a W1 Yokogawa Spinning disk, 50 μm pinhole disk, and a Plan Apo λ 20×/0.75 DIC I objective, or (3) a Nikon Ti2 inverted microscope with a W1 Yokogawa Spinning disk, 50 μm pinhole disk, and a Plan Apo λ 20x/0.8 DIC I objective.

### Image Analysis

#### RPE flatmounts:

RPE flatmounts were quantified as follows. A custom Fiji macro was written to extract four equally sized (~250 μm^2^) boxes containing RPE cell areas from a full-sized RPE flatmount (phalloidin channel) image. The locations of these four boxes were dictated by four lines drawn by the investigator from the outer RPE boundary to the optic nerve head (ONH) in the RPE flatmount, within the GFP+/transduced region for NaIO_3_ images or anywhere around the flatmount for saline images. The midpoints of these four lines were automatically calculated and used to define one corner of each of the four box placements. Care was taken by the investigator to ensure that these lines were drawn such that boxes would be roughly equally spaced around the flatmount and regions of out of focus imaging or tissue processing damage were avoided. The number of RPE cells remaining in each of the four regions placed by the macro was manually counted by the investigator using a built-in Cell Counter feature in Fiji. The median RPE count from these four regions was then calculated and reported. In few cases, a boxed region was suboptimal for manual quantification due to out of focus imaging or weak staining and was thus omitted from the median calculation.

When adult mouse injections were performed, partial transduction was the expected result, as shown by GFP. In these cases, for intra-eye injections (Figure 5), this macro was run twice on the same flatmount, once with four lines drawn from the outer edges to the ONH traversing a *transduced* area of the RPE flatmount, and once with four lines drawn from the outer edges to the ONH traversing an *untransduced* area of the RPE flatmount, using the GFP channel to define transduced areas. The median RPE counts from the transduced and untransduced areas were calculated and reported. For control vector injected RPEs that showed RPE cell death in the central region of the flatmount, “transduced area” was defined by identifying H2B-GFP+ peripheral (ciliary margin-adjacent) RPE cells which were not killed by NaIO_3_ injection. Lines were drawn starting from RPE edges directly adjacent to those surviving peripheral GFP+ cells to the ONH in the center of the flatmount.

#### Retinal flatmounts:

Cone counts for rats and mice injected with saline from the Figure 1B/E and Figure S1B/E cohorts of animals were previously published by our group to provide an estimate of cone density in wild type animals in a concurrent study.^72^ Retinal flatmounts for rats and mice in this study were quantified as follows. For flatmounts with full transduction, a custom Fiji macro extracted four equally sized (~250 μm^2^) boxes containing labeled cones from a given full-sized retinal flatmount image. The locations of these four boxes were placed at the midpoints of four separate lines drawn by the investigator from the outer retinal boundary to the ONH in the full-sized retinal flatmount image itself. Instead of manually counting cells in each of the four boxes drawn on the flatmount, the macro automatically thresholded and counted the number of puncta in each square box and produced four counts (one from each box). The median of these four counts was reported for cone survival assays.

When there was partial transduction, the sample was either excluded (see Sample Exclusion Criteria below), or the macro was run twice on the same flatmount, once with four lines drawn from the outer edges to the ONH traversing a *transduced* area of the retinal flatmount, and once with four lines drawn from the outer edges to the ONH traversing an *untransduced* area of the retinal flatmount. This was done primarily for adult mouse subretinal injections where partial transduction was the expected injection outcome. Transduced areas were marked by AAV8/RedO-H2B-GFP co-injection, which labels cones in retinal flatmounts.

#### Whole Eye or Eyecup Sections:

Spider plots were generated for ONL thickness measurements. Single ONL thickness measurements were made on either side of the ONH at the indicated distances away from the ONH. Measurements were not made in areas of the sections that were untransduced, based upon the GFP+ signal. If tissue damage or sectioning/processing issues precluded a measurement at a particular distance from the ONH, that measurement wasn’t collected. Cryosections and/or paraffin H&E sections were used for analysis (as noted in the Figure legends). OlyVIA visualization software was used to make these thickness measurements.

For Figure 8F only, measurements of ONL thickness were made using ImageJ/Fiji in midperipheral regions (~1–2 mm away from the ONH in rats) of H&E stained paraffin sections. A total of 1–2 sections were analyzed per eye and the average ONL thickness was plotted.

### Sample Exclusion Criteria

Flatmounts were excluded from both eyes of animals that appeared to have an IP injection issue or general lack of NaIO_3_ induced damage in its control AAV/NaIO_3_ injected eye. Some animals were missing a retina, RPE, or section quantification because the tissue in that eye was too torn or damaged post-dissection to flatmount/quantify, or there was an antibody/IHC staining or imaging issue (rare). Lastly, for rats treated with NaIO_3_ for which the NRF2 eye was not completely transduced (as determined by inspection of RedO-H2B-GFP signal in the retinal flatmount), those retinal flatmounts were excluded.

For two mouse ERG cohorts (Figure S4), ERG data was excluded if the histology showed lack of full transduction of the NRF2 eye for a given animal, or if there was no histological data available for reference.

### Statistics

All statistical analyses were performed using GraphPad Prism Version 10, Microsoft Excel, or MATLAB. MATLAB was only used for linear regression analysis of photopic ERG data. For all figures, n refers to individual eyes. All statistical analyses were performed using a p-value cutoff of 0.05. Any p-values above this cutoff are reported as not significant or n.s. For RPE and cone counts, median cell counts for each eye were plotted as individual data points on graphs, but the error bars shown in each plot represent the mean ± SD.

For comparisons between two independent groups, unpaired Student’s t-tests were used. Paired t-tests were used for comparisons between contralateral eyes from the same animal. When one eye was damaged or otherwise unquantifiable, causing a loss of pairing, these values were ignored for paired t-test calculations. For multiple t-tests analyzed in the same plot, a multiple comparisons correction (e.g., Bonferroni-Dunn) was performed as indicated in the figure legend. For comparisons of three or more groups, a one-way ANOVA with a multiple comparisons test was performed. For experiments involving two factors (e.g., AAV treatment × NaIO_3_ vs. saline in Figure 2), a two-way ANOVA with Šídák’s multiple comparisons test was performed. Scotopic ERG data were also analyzed using a two-way ANOVA with Šídák’s multiple comparisons test. Photopic ERG data across multiple flash intensities were analyzed using linear mixed-effects models. For qPCR analyses, unpaired t-tests (equal variances, 1 tail) were used.

### Linear Mixed-Effect Modeling of Photopic ERG Data

To analyze photopic ERG data shown in Figure 3, we used a mixed-effects linear regression model^50,51^, with fixed effects for the covariates of interest and random effects for each rat, in order to account for the repeated ERG measurements for each animal across flash intensity and time, and for eye-within-rat to account for the fact that each animal served as its own control: one eye received a vector expressing the NRF2 gene and the other a control vector. In Wilkinson notation^76^, the full model was:

(1) $$\text{bWaveAmp}\sim 1+\text{treatment}+\text{time}+\text{logFI}+\left(1|\text{ratID}:\text{eyeID}\right)+\left(1|\text{retID}\right)$$

where *bWaveAmp* is the amplitude of the ERG b-wave (in μV), *treatment* is an indicator variable set to ‘1’ for eyes that received an intraocular injection of NRF2 AAV and ‘0’ for control AAV, *time* represents the time at which the ERG recordings were made (0, 2 or 4 weeks after the IP injection of NaIO_3_), logFI was the log10 of the flash intensity (in cd·s/m^2^), and the terms (1 | ratID:eyeID) and (1 | ratID) represents the random effect for eye-within-rat and rat, respectively. The full model was fit to the data using the ‘fitlme’ function in MATLAB (MathWorks, Natick, MA), using maximum likelihood. The results are shown in HYPERLINK Table S1.

All the model parameters were highly statistically significant (p < 0.001), and it accounted for just over 50% of the variance, after adjusting for the number of parameters in the model. The magnitude of the *treatment* parameter tells us that, on average, across all flash intensities and timepoints, the NRF2 treated eyes had a b-wave amplitude of about 10 μV greater than the control eyes, with a 95% confidence interval of [4.20, 16.32]. The negative value of the *time* parameter indicates that there was a progressive decrease in b-wave amplitude after NaIO_3_ treatment. We also ran models with interaction terms for ‘*treatment* × *time’* and ‘*treatment* × *logFI*, but neither of the interaction terms was significant. Because we were not interested in the effect of time, *per se*, we also fit a reduced model to the data from each week’s recordings separately. The reduced model was the same as (1) but omitted *time* as a covariate:

(2) $$\text{bWaveAmp}\sim 1+\text{treatment}+\text{logFI}+\left(1|\text{ratID}:\text{eyeID}\right)+\left(1|\text{ratID}\right)$$

As expected, there was no detectable treatment effect at baseline (p = 0.94). The largest treatment effect was at week 2 (Table S2), while the effect at 4 weeks was smaller and barely statistically significant (Table S3).

For the week 2 data, the slopes of the NRF2-treated eyes appeared steeper than those of the controls (Figure 3B) so we fit an additional model that included an interaction term for *treatment* and *logFI*:

(3) $$\text{bWaveAmp}\sim 1+\text{treatment}+\text{logFI}+\text{treatment}:\text{logFI}+\left(1|\text{ratID}:\text{eyeID}\right)+\left(1|\text{ratID}\right)$$

The interaction term for the 2-week data was statistically significant (7.25 μV / decade flash intensity, 95% CI [1.0, 13.5], p = 0.023; model adjusted R^2^ = 0.72), indicating that the eyes treated with NRF2 had a steeper slope to the relationship between b-wave amplitude and flash intensity than did control eyes (NRF2, slope = 29.2 μV / decade flash intensity; Ctrl, slope = 21.9 μV / decade flash intensity). The difference in slopes is shown by the heavy lines representing the model fits (Figure 3B). The interaction term was not significant for the 4-week data (p = 0.87; Figure 3C).

Because the treatment-vs-control data were paired within rat (i.e. for each rat that received an IP injection of the sodium iodate toxin, one eye received NRF2 and the other eye received a null construct), we further analyzed the *differences* between eyes, which allowed us to remove sources of *shared variability* due to differences among rats in the response to NaIO_3_ treatment. Such sources of shared variability might include procedural variability, such as the actual amount of toxin injected into a given rat, as well as biological variability in each rat’s weight, metabolism and RPE response to the toxin. Such shared variability is evident in Figure 3, particularly for the week 2 data, where the values for the 2 eyes in each rat are connected by a line. We also plotted these between-eye differences for each rat across different flash intensities (Figure S10A). The plots of the 0-week data are randomly distributed about the line y = 0, which is the null value for the paired comparisons. For the 2- and 4-week data, the fact that the preponderance of the lines are above y = 0 is indicative of the treatment effect.

We quantified this treatment effect by fitting a simplified mixed effects model to the data in the above plot:

(4) $$\text{bWaveAmpDiff}\sim 1+\text{logFI}+\left(1|\text{ratID}\right)$$

Where the dependent variable is now the within-rat difference in b-wave ERG amplitude between the treatment and control eyes. The random effect for ratID allows each rat to have its own y-intercept (as for the previous mixed effect models). The results of this fit are shown in Table S4.

In this case, because we are operating on differences, both the y-intercept and the slope (*logFI* coefficient) are of interest: the y-intercept represents a significant offset due to the treatment, and the positive coefficient for *logFI* (slope) indicates that the effect of the treatment increases with flash intensity. In fact, the values for these two coefficients are identical to the values for the *treatment and* interaction term (*treatment* × *logFI*) in model (3). However, because the within-animal subtraction effectively removed a source of shared variability, the standard errors for the coefficients in model (4) are smaller than those of model (3), resulting in lower pvalues: 0.0012 vs. 0.023, for the slope of model (4) vs. the interaction term of model (3).

Importantly, all of the above analyses are in agreement and consistent with the interpretation of a large protective effect of the NRF2 treatment at 2 weeks and a more moderate effect at 4 weeks, both of which are highly statistically significant.

Finally, we performed two diagnostic tests of the model fits, which, taken together, indicate that our use of the linear model was appropriate. A plot of the raw residuals vs. the fitted values shows reasonable homoscedasticity and linearity, while the Q-Q plot displays the normality of the residuals (Figure S10B).

## Supplementary Material

1

## Data Availability

Raw data supporting the findings of this study can be made available upon request. MATLAB code and raw data used to generate the linear mixed-effects models of photopic ERG data are available here: https://github.com/rickborn/NaIO3-Paper.git
