# AAV NRF2 gene therapy preserves retinal structure and function in rodent models of oxidative damage

**DOI:** 10.1016/j.ymthe.2026.03.001

**Published:** 2026-03-22

**Authors:** Apolonia Gardner, Shuai Wang, Adam Daniels, Dan Li, Christine Wu, Lucas Lin, Christin Hong, Sophia R. Zhao, Richard T. Born, Kamil Kruczek, Genevieve Weist, Laura Barrio Real, Joan Wicks, Mohamad Nayal, Ashley Carter, Christine Ott, Virginia Haurigot, Constance L. Cepko

## Main text

(Molecular Therapy *34*, 3245–3269; June 2026)

In the originally published version of this article, the author list mistakenly appeared out of order; specifically, Richard T. Born appeared later in the author list than intended. The corrected author list appears here and has been revised in the published article. The authors regret this error.

