# Supplementary material for: AAV NRF2 Gene Therapy Preserves Retinal Structure and Function in Rodent Models of Oxidative Damage
Source: Mol Ther. Author manuscript; Available in PMC 2026 Jun 10. (PMC13238944; doi:10.1016/j.ymthe.2026.02.005)
Supplement: 1 [file NIHMS2151801-supplement-1.pdf]

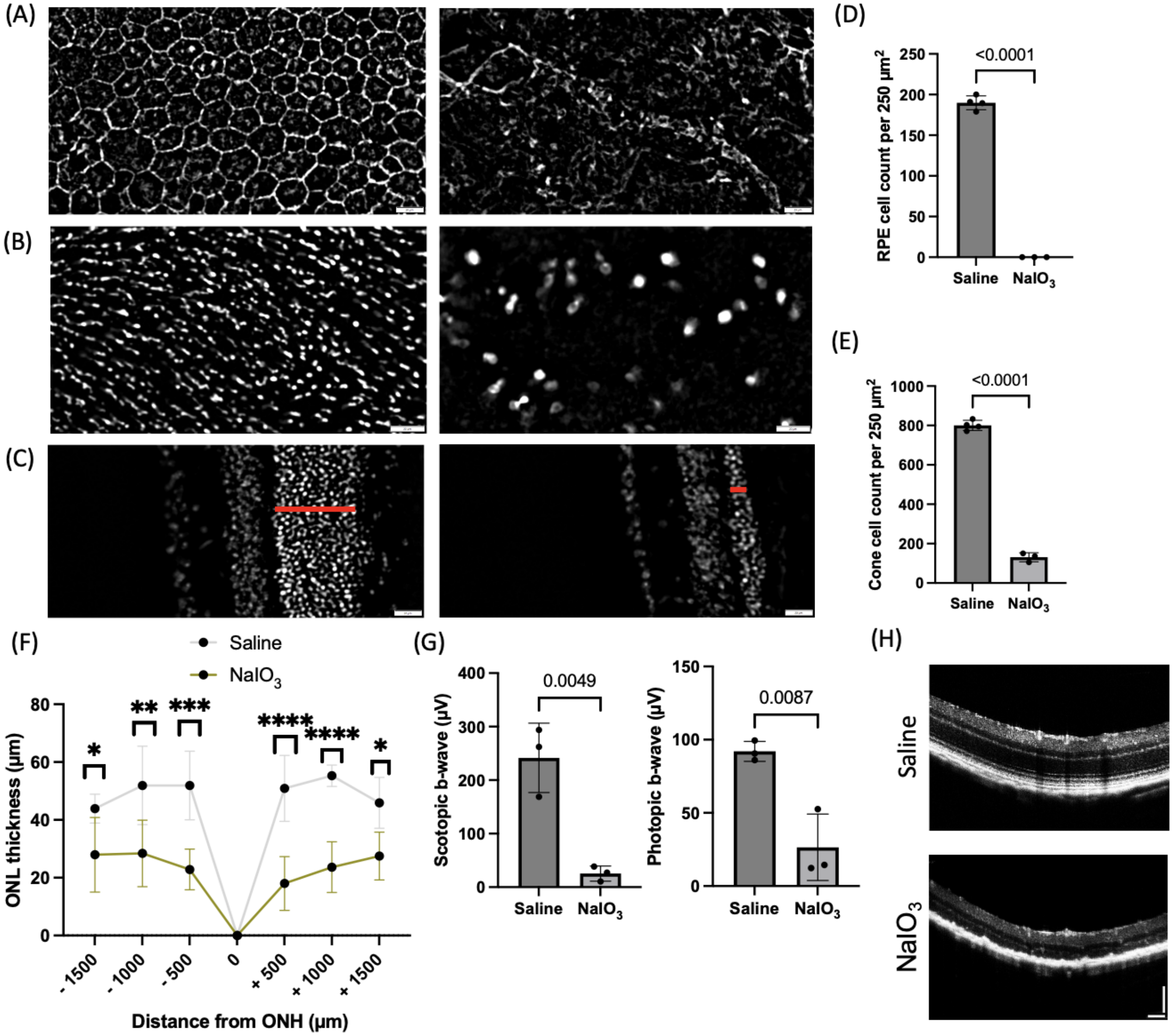

Figure S1: Assessment of RPE and retinal histology after IP injection of $NaIO_3$ or saline in mice. C57BL/6J mice were IP injected with saline or $NaIO_3$ at ~6-8 weeks of age. Tissue was harvested 4-7 weeks post IP injection.

(A) Representative RPE flatmounts (central region) from a saline (left) or $NaIO_3$ (right) IP-injected mouse. Flatmounts were stained with phalloidin (white). Scale bar is 20 microns.

(B) Representative retinal flatmounts (central region) from a saline (left) or $NaIO_3$ (right) IP-injected mouse. Flatmounts were stained with an antibody to CAR (white). Scale bar is 20 microns.

(C) Representative eyecup cryosections (mid-peripheral region) from a saline (left) or $NaIO_3$ (right) IP-injected mouse. Sections were stained with DAPI (white). Red bar indicates the ONL. Scale bar is 20 microns.

(D) Quantification of the number of RPE cells (see Methods, n=3-4 eyes per group, mean ± SD, $p<0.0001$; unpaired T test).

(E) Quantification of the number of cones (see Methods, n=3-4 eyes per group, mean ± SD, $p<0.0001$; unpaired T test).

(F) ONL thickness spider plot for eyecup cryosections (n=4-7 per group, mean ± SD, left to right on plot: *p=0.0495, **p=0.0026, ***p=0.0001, ****p<0.0001, *p=0.0158, two-way ANOVA with Šídák's multiple comparison test).

(G) Scotopic ERG b-wave amplitudes (at a 0.1 cd.s/m^2 flash stimulus, left) and photopic ERG b-wave amplitudes (at a 10 cd.s/m^2flash stimulus, right) for mice injected with saline or $NaIO_3$ were collected 3-4 weeks post IP injection (n=3 mice per group, mean ± SD, p=0.005 for scotopic and p=0.009 for photopic, unpaired T test for each plot).

(H) Representative OCT images for a saline IP-injected mouse (top panel) or an $NaIO_3$ IP-injected mouse (bottom panel) at 4 weeks post IP injection (n=3 mice per group, scale bar is 100 microns).

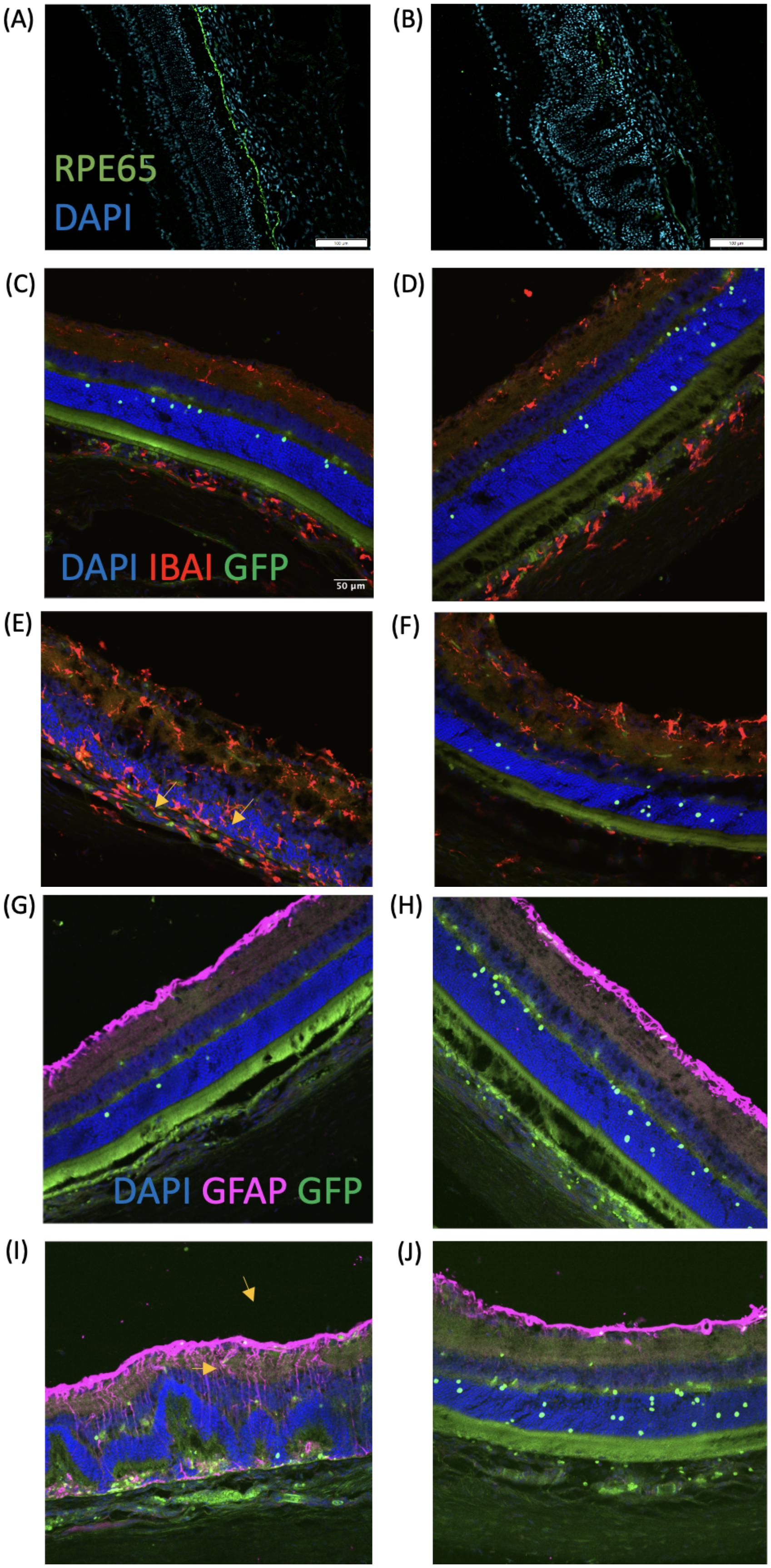

Figure S2: RPE65, IbaI and GFAP staining on rat sections. For panels A-B, Sprague-Dawley rats were IP injected with saline or $NaIO_3$ at 6-8 weeks of age. Tissue was harvested 4-5 weeks post IP injection. RPE65 staining is shown in green. DAPI staining is shown in blue. Scale bar is 100 microns. For remaining panels, Sprague-Dawley rats were injected subretinally at birth with 2e9 vg AAV8/Best1-NRF2 + 2e7 vg AAV8/RedO-H2B-GFP in one eye or 2e9 vg AAV8/Best1-6xSTOPmutGFP + 2e7 vg AAV8/RedO-H2B-GFP in the other, as in Figures 2D-2E. At 6-6.5 weeks of age, rats were IP injected with saline or $NaIO_3$. Eyes were harvested 4-4.5 weeks post IP injection. Channels include: blue (DAPI), green (GFP), red (IbaI) and far red (GFAP). Scale bar is 50 microns.

(A) Representative eyecup sections from rats IP injected with saline.

(B) Representative eyecup sections from rats IP injected with $NaIO_3$.

(C) Representative control AAV-injected eye from animals injected with saline.

(D) Representative AAV8/Best1-NRF2 injected eye from animals injected with saline.

(E) Representative control AAV-injected eye from animals injected with $NaIO_3$. Orange arrows indicate myeloid cells stained by IbaI.

(F) Representative AAV8/Best1-NRF2 injected eye from animals injected with $NaIO_3$.

(G) Representative control AAV-injected eye from animals injected with saline.

(H) Representative AAV8/Best1-NRF2 injected eye from animals injected with saline.

(I) Representative control AAV-injected eye from animals injected with $NaIO_3$. Orange arrows indicate GFAP+ cells and their processes.

(J) Representative AAV8/Best1-NRF2 injected eye from animals injected with $NaIO_3$.

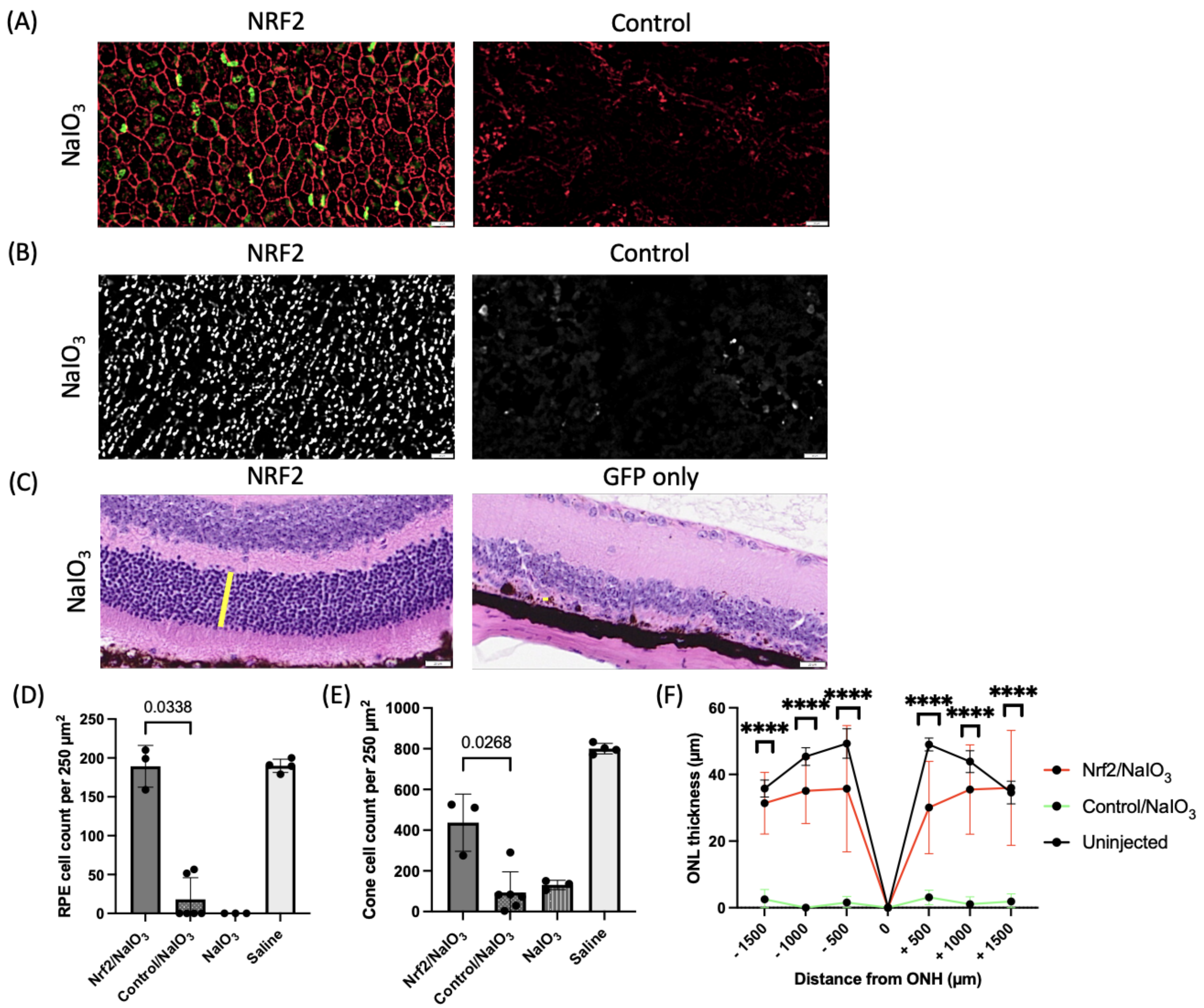

Figure S3: Assessment of AAV8/Best1-NRF2 on RPE and retinal histology in mice. For panels A/B/D/E, mice were injected at birth with 4e8 vg AAV8/Best1-NRF2 + 2e8 vg AAV8/RedO-H2B-GFP in one eye or 4e8 vg AAV8/Best1-6xSTOP-mutGFP control vector + 2e8 vg AAV8/RedO-H2B-GFP in the contralateral eye and IP injected with $NaIO_3$ at 8-10 weeks of age. Tissues were harvested 12-13 weeks post IP injection. For panels C/F, mice were injected at birth with 4e8 vg AAV8/Best1-NRF2 + 1e7 vg AAV8/RedO-H2B-GFP in one eye or 1e7 vg AAV8/RedO-H2B-GFP in the contralateral eye and IP injected with $NaIO_3$ at 6-6.5 weeks of age. Eyes were harvested 12-13 weeks post IP injection and processed for hematoxylin & eosin staining. N refers to individual eyes.

(A) Representative RPE flatmounts (central region) stained with phalloidin (red). Scale bar is 20 microns.

(B) Representative retinal flatmounts (central region) stained with an antibody to CAR (white). Scale bar is 20 microns.

(C) Representative eyecup sections (mid-peripheral region) stained with H&E. Yellow bar indicates the ONL. Scale bar is 20 microns.

(D) Quantification of the number of RPE cells (n=3 NRF2/$NaIO_3$ flatmounts, n=6 Control/$NaIO_3$ flatmounts, mean ± SD, p=0.03; paired T test). Data from n=3-4 saline and $NaIO_3$ samples are replotted here from Figure S1 for reference (max age of Fig S1 references: ~15 weeks; max age of Fig S3 samples: ~23 weeks).

(E) Quantification of the number of cones (n=3 for NRF2/$NaIO_3$ flatmounts, n=6 Control/$NaIO_3$ flatmounts, mean ± SD, p=0.03; paired T test). Data from n=3-4 saline and $NaIO_3$ samples are replotted here from Figure S1 for reference (max age of Fig S1 references: ~15 weeks; max age of Fig S3 samples: ~23 weeks).

(F) ONL thickness spider plot for the paraffin H&E sections described in (C). As a healthy eye control, uninjected C57BL/6J mice (~7-9 weeks old) were harvested and processed with H&E staining (n=4-6 per group, mean ± SD, p<0.0001 for NRF2/$NaIO_3$ vs. Control/$NaIO_3$ comparison, two-way ANOVA with Šídák's multiple comparison test).

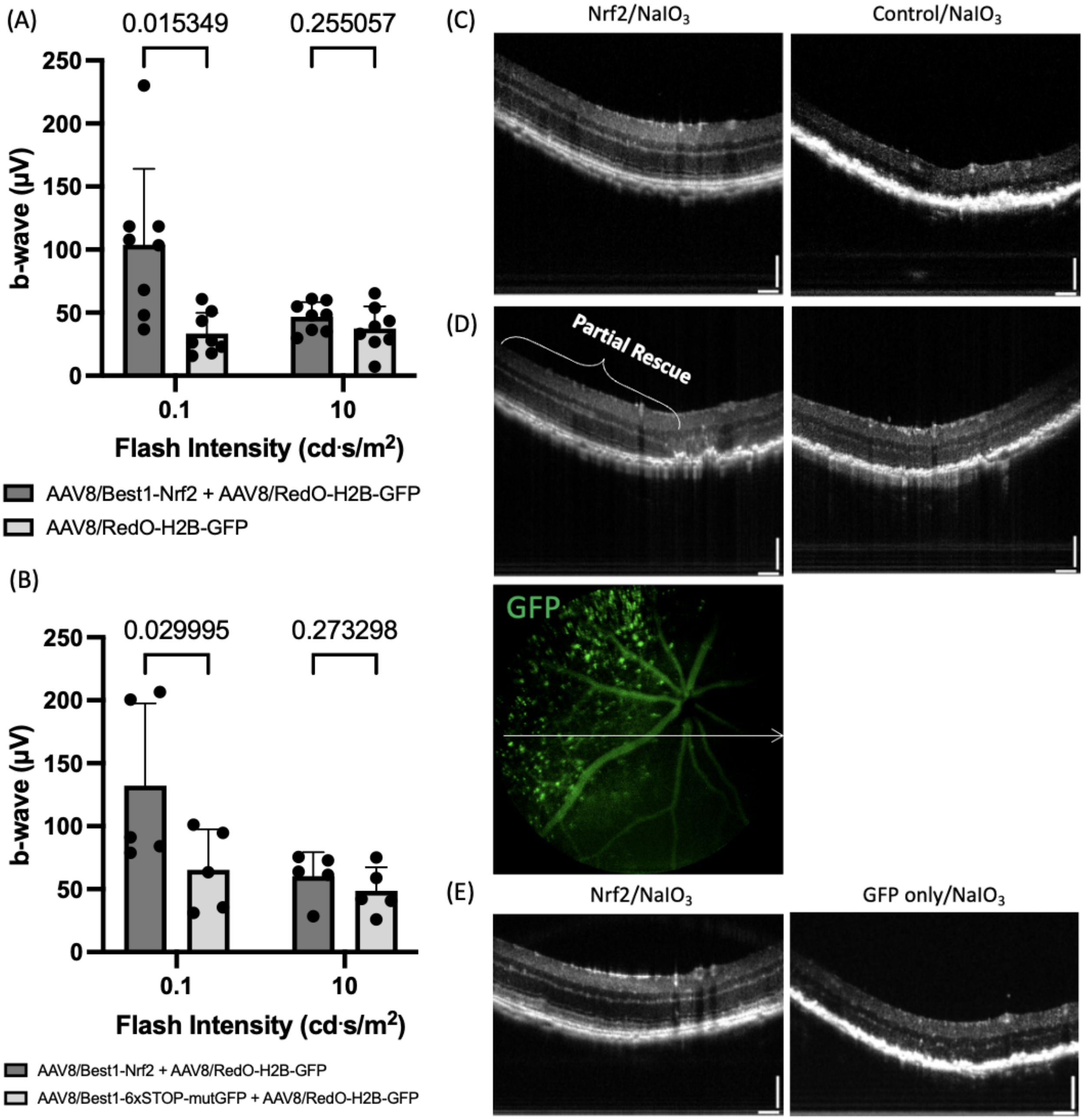

Figure S4: Assessment of AAV8/Best1-NRF2 on in-life visual function and retinal structure in two mouse cohorts. C57BL/6N mice were injected at birth with 4e8 vg AAV8/Best1-NRF2 + 1e7 vg AAV8/RedO-H2B-GFP in one eye or only 1e7 vg AAV8/RedO-H2B-GFP in the contralateral eye and IP injected with $NaIO_3$ at 6-7 weeks of age (panels A and E). Alternatively, C57BL/6N mice were injected at birth with 4e8 vg AAV8/Best1-NRF2 + 2e8 vg AAV8/RedO-H2B-GFP in one eye or 4e8 vg AAV8/Best1-6xSTOP-mutGFP control vector + 2e8 vg AAV8/RedO-H2B-GFP in the contralateral eye and IP injected with $NaIO_3$ at 8-10 weeks of age (panels B-D). Scotopic ERG used a flash stimulus of 0.1 cd.s/m^2, and photopic ERG used a flash stimulus of 10 cd.s/m^2. ERG b-wave amplitudes are plotted. Images were acquired ~4 weeks post IP injection. All mice received an $NaIO_3$ IP injection.

(A) ERG data was acquired ~3-4 weeks post IP injection (n=8 mice, mean ± SD, p=0.02 for scotopic comparison, multiple paired T tests with Bonferroni-Dunn multiple comparisons correction).

(B) ERG data was acquired ~4-5 weeks post IP injection (n=5 mice, mean ± SD, p=0.03 for scotopic comparison, multiple paired T tests with Bonferroni-Dunn multiple comparisons correction).

(C) Representative OCT images (full AAV8/Best1-NRF2 transduction).

(D) Representative OCT images (partial AAV8/Best1-NRF2 transduction detectable by OCT and the GFP+ area in the fluorescent fundus image).

(E) Representative OCT images (full AAV8/Best1-NRF2 transduction).

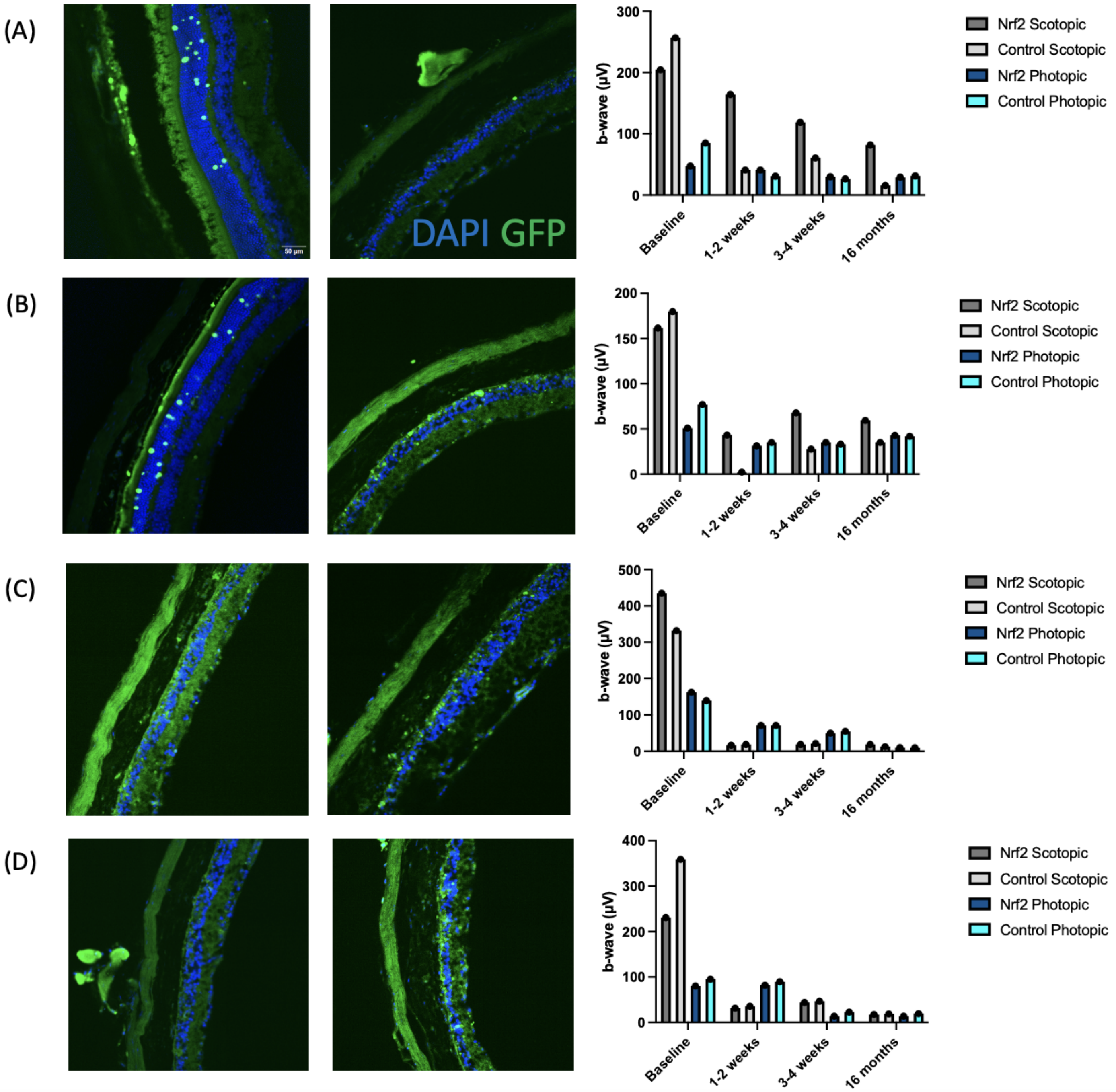

Figure S5. Assessment of AAV8/Best1-NRF2 on retinal histology and function in mice ~16 months post subretinal injection. C57BL/6N mice were injected at birth with 4e8 vg AAV8/Best1-NRF2 + 1e7 vg AAV8/RedO-H2B-GFP in one eye or only 1e7 vg AAV8/RedO-H2B-GFP in the contralateral eye and IP injected with $NaIO_3$ at 6-7 weeks of age. Eyes were harvested at ~16 months of age for cryosectioning and ONL thickness analysis. Left image: left eye (injected with 4e8 vg AAV8/Best1-NRF2 + 1e7 vg AAV8/RedO-H2B-GFP). Right image: right eye (injected with 1e7 vg AAV8/RedO-H2B-GFP). DAPI staining is shown in blue and H2B-GFP labeling is shown in green. Each panel (A-D) represents a different mouse. Scale bar is 50 microns. ERG data from each animal at various timepoints are provided on the right.
(A) Representative left/right eyecup sections and ERG data from male #1.
(B) Representative left/right eyecup sections and ERG data from male #2.
(C) Representative left/right eyecup sections and ERG data from male #3. The left eye injected with AAV8/Best1-NRF2 appeared not well transduced.
(D) Representative left/right eyecup sections and ERG data from male #4. The left eye injected with AAV8/Best1-NRF2 appeared not well transduced.

(A)

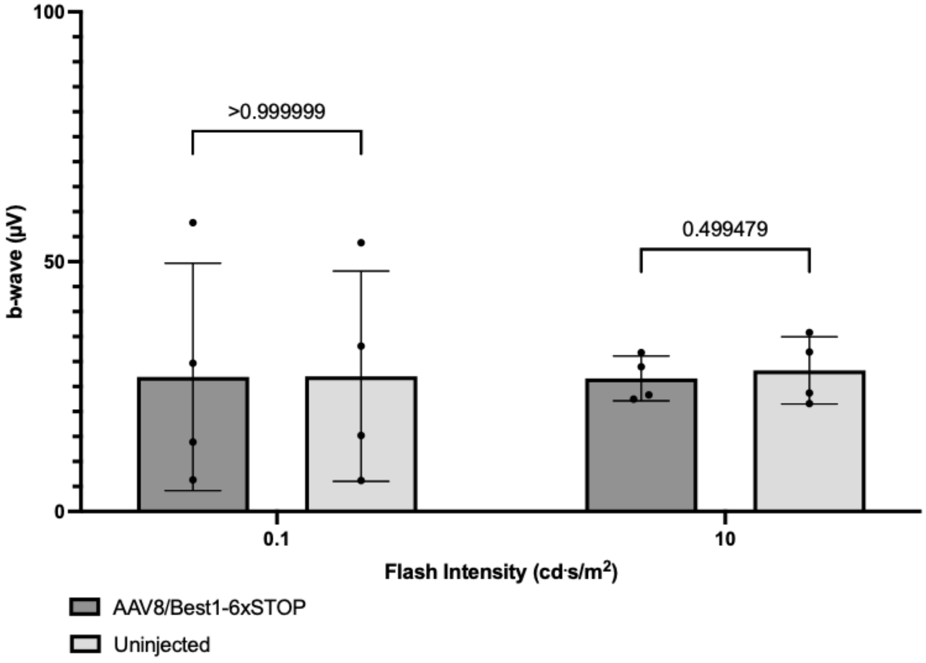

(B)

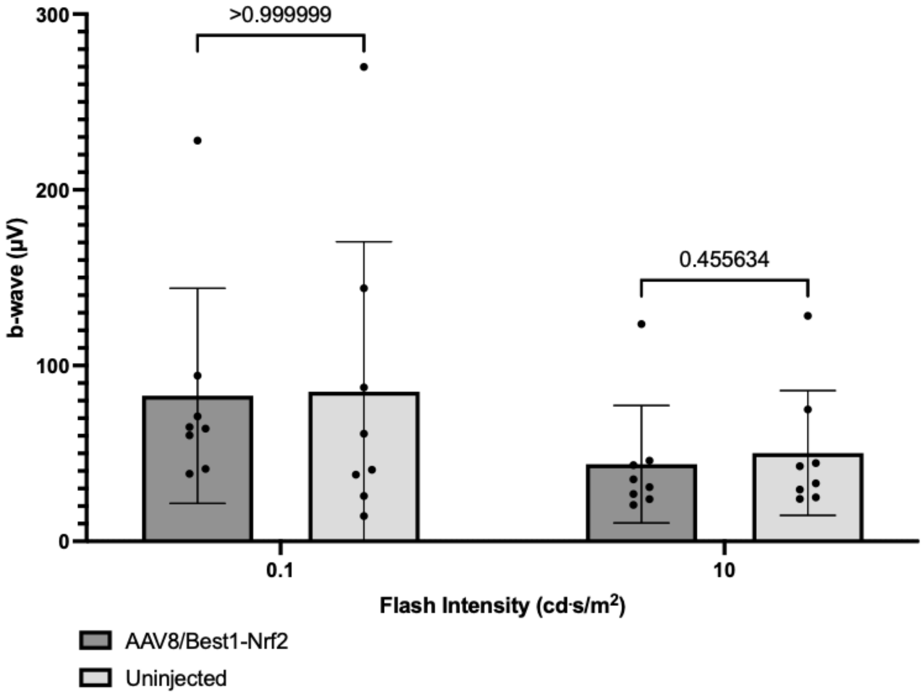

Figure S6: Assessment of AAV8/Best1-NRF2 on visual function in mice subretinally injected as adults. C57BL/6J mice were subretinally injected at 6-13 weeks of age with 2e9 vg AAV8/Best1-6xSTOP control vector + 2e9 vg AAV8/RedO-H2B-GFP in the right eye, with the left eye uninjected (panel A). Alternatively, C57BL/6J mice were subretinally injected at 6-13 weeks of age with 2e9 vg AAV8/Best1-NRF2 + 2e9 vg AAV8/RedO-H2B-GFP in the right eye, with the left eye uninjected (panel B). Animals were IP injected with $NaIO_3$ at 1-2 weeks post subretinal injection. ERG data were collected 6-7 weeks post IP injection. Scotopic ERG used a flash stimulus of 0.1 cd.s/m^2, and photopic ERG used a flash stimulus of 10 cd.s/m^2. ERG b-wave amplitudes are plotted.
(A) ERG data (n=4 mice, mean ± SD, multiple paired T tests with Bonferroni-Dunn multiple comparisons correction).
(B) ERG data (n=8 mice, mean ± SD, multiple paired T tests with Bonferroni-Dunn multiple comparisons correction).

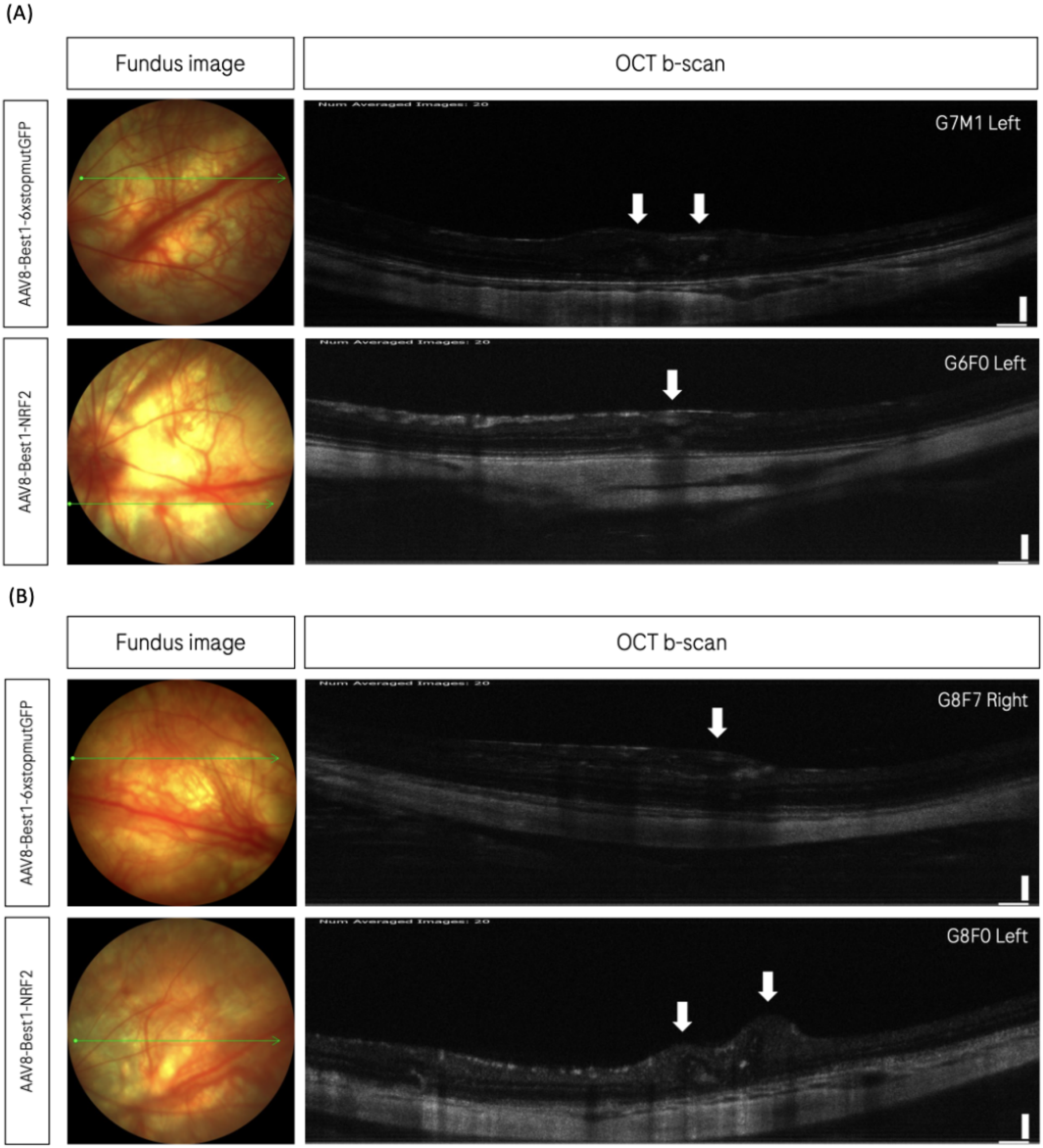

Figure S7: Representative OCT images showing localized areas of retinal disruption. Tolerability study rats were injected with the indicated dose of AAV8/Best1-NRF2 or AAV8/Best1-6xSTOP-mutGFP in contralateral eyes at birth. No IP injections were performed. OCTs were collected at 9-10 months of age and representative OCT scans are shown. Arrows indicate localized areas of retinal disruption in OCT scans.

(A) OCT scans from rats injected with 4e8 vg of AAV8/Best1-NRF2 or AAV8/Best1-6xSTOP-mutGFP.

(B) OCT scans from rats injected with 2e9 vg of AAV8/Best1-NRF2 or AAV8/Best1-6xSTOP-mutGFP.

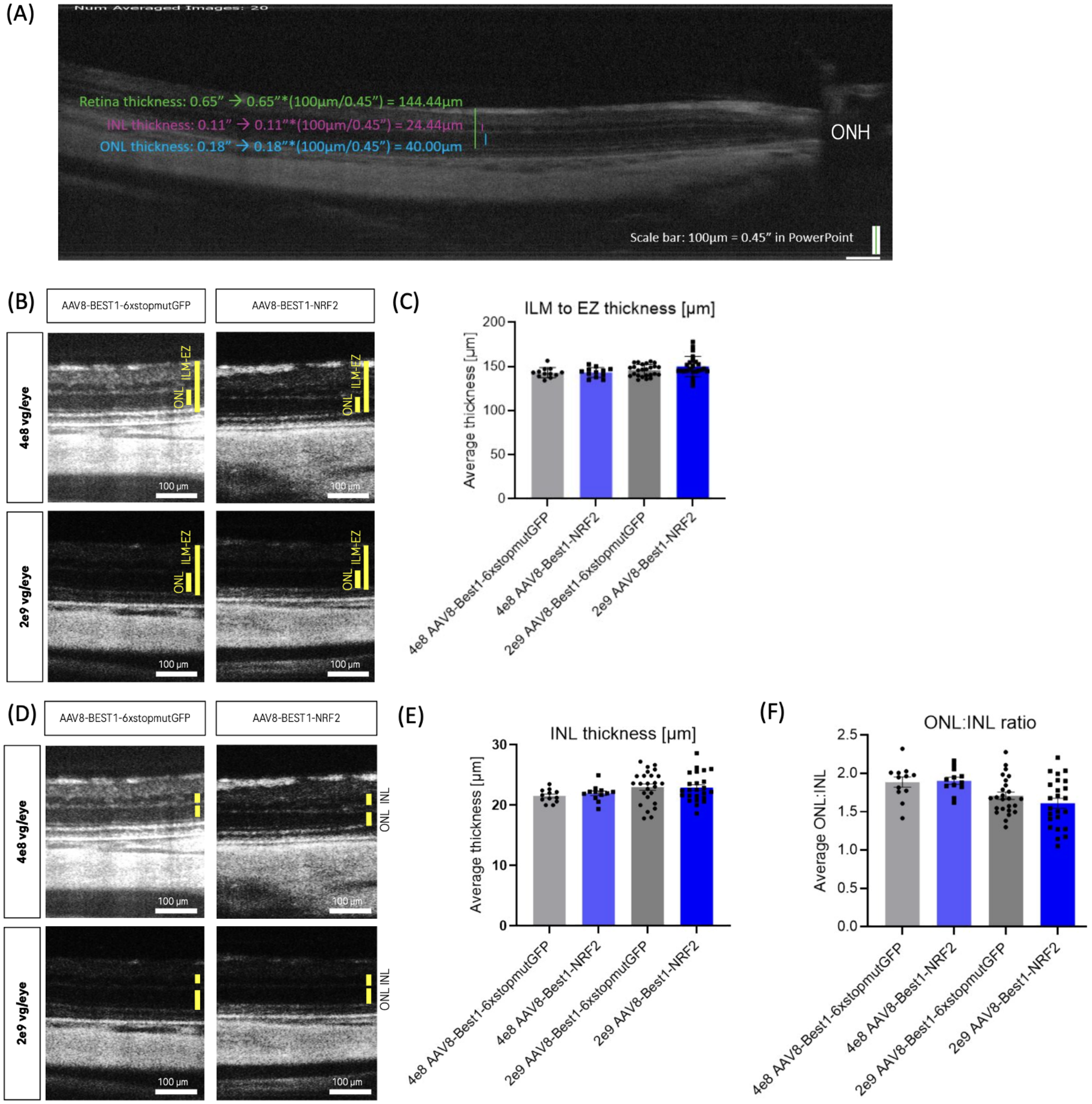

Figure S8: ILM-EZ thickness, INL thickness, and ONL:INL ratio quantification results from rat tolerability OCT images. Tolerability study rats were injected with the indicated dose of AAV8/Best1-NRF2 or AAV8/Best1-6xSTOP-mutGFP in contralateral eyes at birth. No IP injections were performed. OCT images were collected at 9-10 months of age and representative OCT scans are shown. Neurosensory retinal layer thicknesses were measured from the inner limiting layer (ILM) to the ellipsoid zone (EZ). ONL and INL thickness measurements were taken for all available rat tolerability OCT images. Ratio of ONL:INL thickness was calculated to normalize for regional differences in retinal thickness that may affect individual measurements of ONL thickness.

(A) Diagram of the methodology used for OCT image quantification for rat tolerability data.
(B) Representative OCT images where ONL and ILM-EZ measurements were taken.
(C) Quantification of ILM-EZ thickness.
(D) Representative OCT images where ONL and INL measurements were taken. The same scans are used as examples in panels (B) and (D) in this figure.
(E) Quantification of INL thickness.
(F) Quantification of ONL:INL ratio.

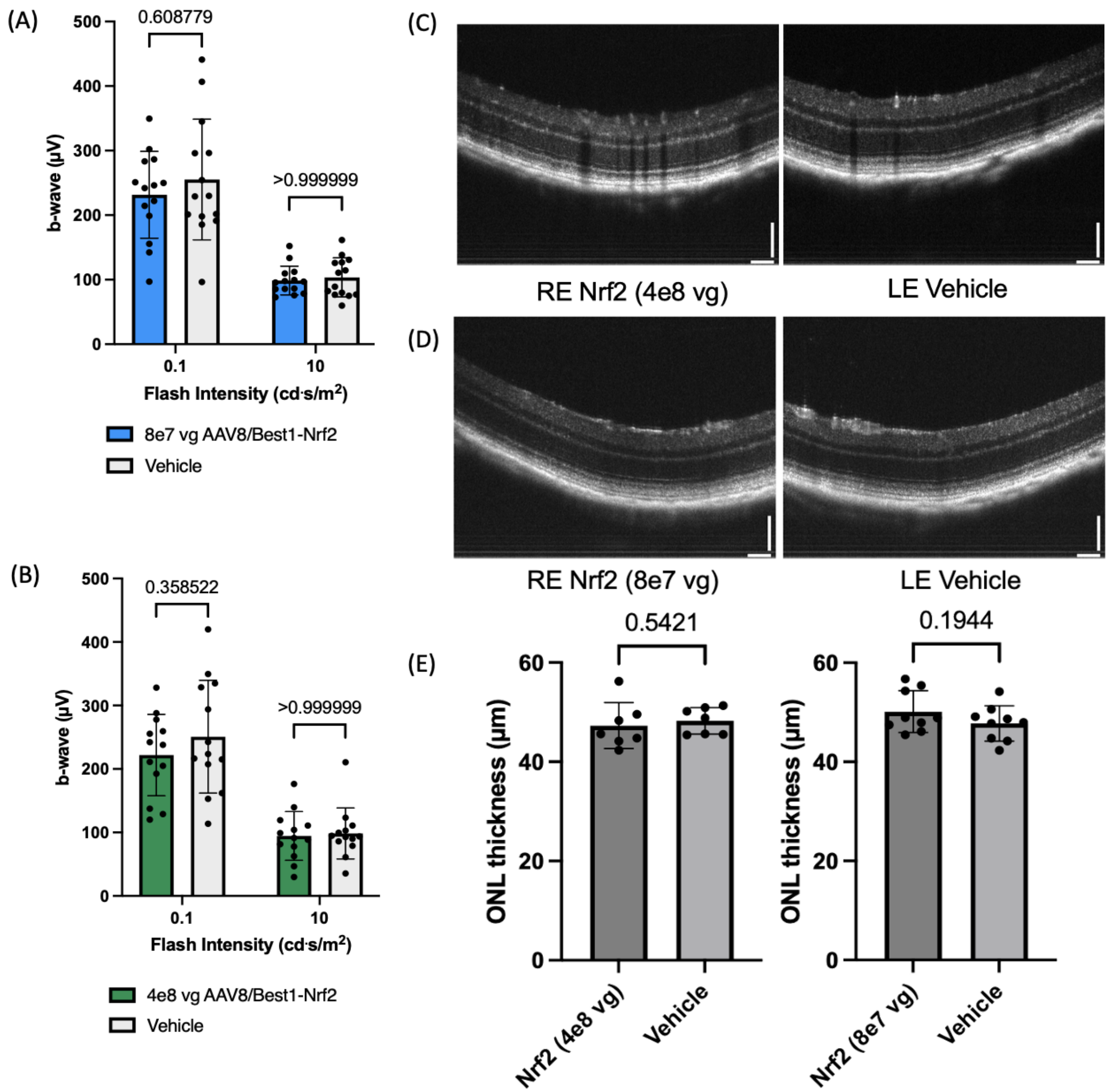

Figure S9: Tolerability assessments for AAV8/Best1-NRF2 in mice. Tolerability study mice were injected with the indicated dose of AAV8/Best1-NRF2 or PBS vehicle control in contralateral eyes at birth. No IP injections were performed. Scotopic ERG used a flash stimulus of 0.1 cd.s/m^2, and photopic ERG used a flash stimulus of 10 cd.s/m^2. B-wave amplitudes are plotted. ERG data was collected from mice at 1-2 months of age. OCT images were collected from mice at 8-9 months of age.

(A) ERG data from C57BL/6N mice injected with AAV8/Best1-NRF2 (8e7 vg) in one eye and a vehicle control (see Methods) in the contralateral eye (n=14 mice, mean ± SD, multiple paired T tests with Bonferroni-Dunn multiple comparisons correction).

(B) ERG data from C57BL/6N mice injected with AAV8/Best1-NRF2 (4e8 vg) in one eye and a vehicle control in the contralateral eye (n=13 mice, mean ± SD, multiple paired T tests with Bonferroni-Dunn multiple comparisons correction).

(C) Representative OCT images from C57BL/6J mice injected with 4e8 vg AAV8/Best1-NRF2 in one eye and a vehicle control in the contralateral eye (n=7 mice). Scale bar is 100 microns.

(D) Representative OCT images from C57BL/6J mice injected with 8e7 vg AAV8/Best1-NRF2 in one eye and a vehicle control in the contralateral eye (n=9 mice). Scale bar is 100 microns.

(E) Quantification of ONL thickness in OCT images from the mice described in panels C-D (n=7 for left plot, n=9 for right plot, mean ± SD, paired t-test performed for each plot).

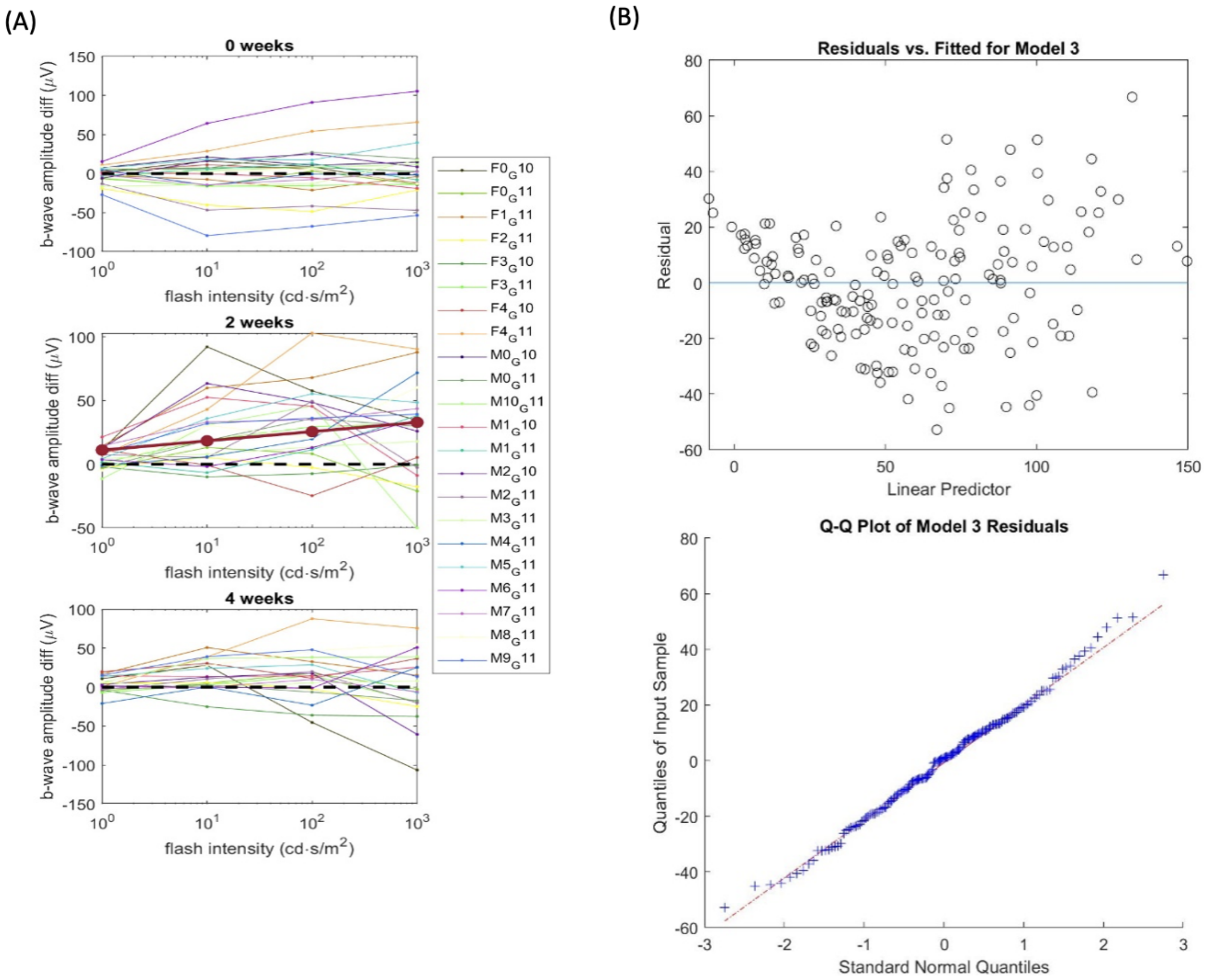

Figure S10. Additional analyses of Rat Photopic ERG data. Rats were subretinally injected at birth with 2e9 vg AAV8/Best1-NRF2 + 2e9 vg AAV8/RedO-H2B-GFP in one eye or 2e9 vg AAV8/Best1-6xSTOPmutGFP + 2e9 vg AAV8/RedO-H2B-GFP in the other. At P43, rats were IP injected with $NaIO_3$. Eyes were harvested at 5-6 weeks post IP injection. ERG data was collected at three timepoints: baseline (before IP injection), 2 weeks post IP injection, and 4 weeks post IP injection of saline or $NaIO_3$. Multiple photopic flash intensities ranging from 10^0 – 10^3 cd.s/m^2 were collected for each rat at each timepoint.

(A) Between-eye b-wave amplitude (NRF2 eye – control eye) difference plots for the cohort of rats described above (and shown in Figure 3) at baseline, 2 weeks, and 4 weeks post IP injection. Each colored line represents an individual rat in the cohort. The black dashed line is the y=0 line. The legend on the right indicates the rat ID.

(B) Regression diagnostics for model (3) fit to the 2-week data. (Top) Plot of residuals vs. linear predictors. (Bottom) Q-Q plot of model 3 residuals. The raw data, analysis code and results are available at: https://github.com/rickborn/NaIO3-Paper.git

Table S1. Results for full model (1). Model AIC = 4932; adjusted R^2 = 0.52

Name	Estimate	Std. Error	t-Stat	DF	p Value	95% CI (lower)	95% CI (upper)
y-intercept	29.57	4.20	7.03	500	6.65×10^{-12}	21.31	37.84
treatment	10.26	3.08	3.33	500	9.41×10^{-4}	4.20	16.32
time	-4.81	0.84	-5.70	500	2.02×10^{-8}	-6.47	-3.15
logFI	24.65	1.22	20.25	500	5.23×10^{-67}	22.25	27.04

Table S2. Results for reduced model (2) fit to 2-week data. Model AIC = 1588; adjusted R^2 = 0.71

Name	Estimate	Std. Error	t-Stat	DF	p Value	95% CI (lower)	95% CI (upper)
y-intercept	9.17	5.21	1.76	165	0.080	-1.11	19.45
treatment	21.95	4.09	5.36	165	2.71×10^{-7}	13.87	30.02
logFI	25.57	1.62	15.83	165	6.37×10^{-35}	22.38	28.76

Table S3. Results for reduced model (2) fit to 4-week data. Model AIC = 1570; adjusted R^2 = 0.61

Name	Estimate	Std. Error	t-Stat	DF	p Value	95% CI (lower)	95% CI (upper)
y-intercept	12.37	6.59	1.88	157	0.0624	-0.65	25.40
treatment	8.82	4.45	1.98	157	0.0492	0.03	17.60
logFI	24.01	1.99	12.07	157	3.82×10^{-24}	20.08	27.94

Table S4. Results for reduced model (4) fit to 2-week data. Model AIC = 789; adjusted R^2 = 0.33

Name	Estimate	Std. Error	t-Stat	DF	p Value	95% CI (lower)	95% CI (upper)
y-intercept	11.06	5.23	2.12	82	0.0374	0.66	21.47
logFI	7.25	2.17	3.34	82	0.00126	2.93	11.57

Table S5: Quantification of the number of OCT images containing areas of retinal disruption. Tolerability study rats were injected with the indicated dose of AAV8/Best1-NRF2 or AAV8/Best1-6xSTOP-mutGFP in contralateral eyes at birth. No IP injections were performed. OCT images were collected at 9-10 months of age and representative OCT scans are shown in Figure S8. Localized areas of retinal disruption in OCT scans are shown in Figure S7. This table shows the number and % of OCT images containing a retinal disruption.

Quantification type	4e8 vg/eye 6xstopmutGFP	4e8 vg/eye NRF2	2e9 vg/eye 6xstopmutGFP	2e9 vg/eye NRF2
Total number of images examined	108	118	204	199
Retinal disruption present	2	4	10	21
% OCT images with retinal disruption	1.9%	3.4%	4.9%	10.6%

Table S6: Quantification of retinal layer thickness from OCT images

Tolerability study rats were injected with the indicated dose of AAV8/Best1-NRF2 or AAV8/Best1-6xSTOP-mutGFP in contralateral eyes at birth. OCT images were collected at 9-10 months of age. Examples are shown in Figure S8.
This table summarizes the quantification of ILM-EZ, ONL, and INL thicknesses, as well as the ONL:INL ratio (average ± SEM indicated) calculated across all OCT images analyzed. An image showing the areas quantified is shown in Figure S8. For comparison, Figure 8F depicts ONL thicknesses calculated from H&E stained sections of healthy vehicle-injected rats in our study at ~11 months of age: ~35 microns.

	**4e8** vg/eye AAV8-Best1-**6xstopmutGFP**	**4e8** vg/eye AAV8-Best1-**NRF2**	**2e9** vg/eye AAV8-Best1-**6xstopmutGFP**	**2e9** vg/eye AAV8-Best1-**NRF2**
**ILM to EZ thickness**	143.1 ± 1.632	143.3 ± 1.620	145.7 ± 1.335	150.0 ± 2.343
**ONL thickness**	39.53 ± 1.183	40.94 ± 0.8970	37.93 ± 0.9410	34.78 ± 1.032
**INL thickness**	21.53 ± 0.3106	21.96 ± 0.3845	22.98 ± 0.5409	22.91 ± 0.4805
**ONL:INL ratio**	1.886 ± 0.06689	1.904 ± 0.04659	1.706 ± 0.04796	1.610 ± 0.06521

Table S7. Male/female rodent breakdown for main text figures. The number of males and females used in each experiment are shown. Rats were used for Figures 1, 2, 3, 7, and 8. B6J mice were used for Figures 4 and 5. B6N mice were used for Figure 6. *Figure 1 control animals were not included in the counts shown. **Figure 2E counts include Figure 2D counts plus extra cryosectioned animals.

Figure #	Number of males	Number of females
1D	0	4 (2 saline, 2 $NaIO_3$)
1E	4 (all saline)	8 (4 saline, 4 $NaIO_3$)
1F-G	4 (2 saline, 2 $NaIO_3$)	6 (3 saline, 3 $NaIO_3$)
2B-C*	16 (4 saline, 12 $NaIO_3$)	10 (2 saline, 8 $NaIO_3$)
2D	4 (0 saline, 4 $NaIO_3$)	3 (3 saline, 0 $NaIO_3$)
2E*,**	9 (2 saline, 7 $NaIO_3$)	8 (4 saline, 4 $NaIO_3$)
3A-C*	16 (4 saline, 12 $NaIO_3$)	10 (2 saline, 8 $NaIO_3$)
4A*	5 (1 NRF2/$NaIO_3$, 3 control/$NaIO_3$,<br>0 NRF2/saline, 1 control/saline)	12 (3 NRF2/$NaIO_3$, 2 control/$NaIO_3$,<br>4 NRF2/saline, 3 control/saline)
4B*	5 (2 control/$NaIO_3$, 3 control/saline)	10 (3 NRF2/$NaIO_3$, 2 control/$NaIO_3$,<br>3 NRF2/saline, 2 control/saline)
5A	$NaIO_3$ plot: 13 (4 NRF2, 9 control)<br>Saline plot: 2 (0 NRF2, 2 control)	$NaIO_3$ plot: 3 (3 NRF2, 0 control)<br>Saline plot: 5 (3 NRF2, 2 control)
5B	$NaIO_3$ plot: 13 (5 NRF2, 8 control)<br>Saline plot: 5 (2 NRF2, 3 control)	$NaIO_3$ plot: 4 (4 NRF2, 0 control)<br>Saline plot: 5 (3 NRF2, 2 control)
6A	4	0
6B	6	0
7D	7	8
7E	12	15
7F	4	4
7G	0	8
8F	10	9

Table S8. Male/female rodent breakdown for supplemental figures. The number of males and females used in each experiment are shown. Rats were used for Figures S2, S7, S8, and S10. B6J mice were used for Figures S1, S6, S9C-E, and as controls in Figure S3. B6N mice were used for Figures S3, S4, S5, and S9A-B. *Figure S1 control animals were not included in the counts shown.

Figure #	Number of males	Number of females
S1D-E	3 (0 saline, 3 $NaIO_3$)	4 (4 saline, 0 $NaIO_3$)
S1F	6 (4 saline, 2 $NaIO_3$)	4 (2 saline, 2 $NaIO_3$)
S1G	6 (3 saline, 3 $NaIO_3$)	0
S3D-E*	4	2
S3F	0 (+ uninjected control: 1)	5 (+ uninjected controls: 2)
S4A	2	6
S4B	3	2
S6A	4	0
S6B	4	4
S9A	5	9
S9B	5	8
S9C/E	5	2
S9D/E	6	3
S10A	14	8
